# Distinct Resting‐State Connectomes for Face and Scene Perception Predict Individual Task Performance

**DOI:** 10.1002/hbm.70498

**Published:** 2026-03-23

**Authors:** Orhan Soyuhos, Aurelia Scarpa, Daniel Baldauf

**Affiliations:** ^1^ Centre for Mind/Brain Sciences (CIMeC) University of Trento Trento Italy; ^2^ Center for Neuroscience University of California, Davis Davis California USA; ^3^ Department of Psychology University of California, Davis Davis California USA; ^4^ Research Group Health Psychology, Faculty of Psychology and Educational Sciences KU Leuven Leuven Belgium

**Keywords:** face perception, fMRI, functional connectivity, MEG, scene perception

## Abstract

Face and scene perception rely on distinct neural networks centered on the Fusiform Face Area (FFA) and Parahippocampal Place Area (PPA). However, how these regions interact with broader brain networks remains unclear. Using resting‐state fMRI and MEG data, we mapped the spatial and frequency‐specific functional connectivity of the FFA and PPA. We found that the FFA showed predominant fMRI connectivity with lateral occipitotemporal, inferior temporal, and temporoparietal regions, while the PPA connected more strongly with ventral medial visual, posterior cingulate, and entorhinal‐perirhinal areas. MEG analyses further revealed this network segregation was reflected in beta and gamma bands. Importantly, connectome‐based predictive modeling showed that the strength of these intrinsic fMRI connectivity patterns predicted individual reaction times on corresponding face and scene perception tasks. Our findings demonstrate that the FFA and PPA anchor distinct intrinsic networks with unique spatio‐temporal profiles that provide a functional architecture supporting their specialized roles in face and scene perception.

## Graphical abstract

Resting‐state fMRI and MEG functional connectivity reveal that the FFA and PPA anchor distinct spatio‐temporal networks. The strength of this intrinsic architecture shows a double dissociation: FFA‐network connectivity specifically predicts face‐task performance, while PPA‐network connectivity predicts scene‐task performance, linking intrinsic organization to cognitive function.

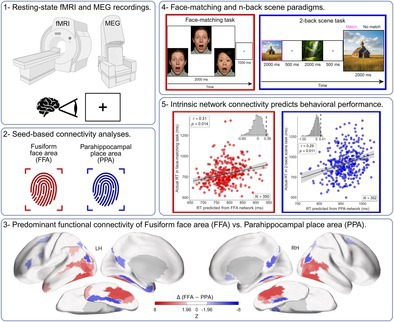

## 1 Introduction

A fundamental principle of cortical organization is the existence of specialized networks dedicated to distinct cognitive domains (Fox and Raichle 2007; Passingham et al. 2002; Power et al. 2011; Thomas Yeo et al. 2011). The processing of faces and scenes represents a canonical example of this functional specialization, supported by distinct yet interconnected neural systems in the human high‐level visual cortex (Dilks et al. 2022; Epstein and Baker 2019; Grill‐Spector and Weiner 2014; Haxby et al. 2000). Specifically, the Fusiform Face Area (FFA) and the Parahippocampal Place Area (PPA) are prominent regions that respond robustly to face and scene stimuli, respectively. The FFA, located in the fusiform gyrus, is critical for face recognition (Kanwisher and Yovel 2006; Kanwisher et al. 1997; Parvizi et al. 2012; Puce et al. 1996; Schalk et al. 2017; Weiner and Zilles 2016), and neuropsychological evidence from patients with prosopagnosia further emphasizes the functional specificity of the FFA (Barton et al. 2002; Damasio et al. 1982; McNeil and Warrington 1993). Similarly, studies in macaques have highlighted the role of face‐selective neurons in the homologous macaque FFA, which exhibit nearly exclusive responses to face stimuli (Tsao et al. 2006). The PPA, situated at the junction of the collateral and anterior lingual sulcus, plays a key role in scene perception and categorization (Dilks et al. 2022; Epstein and Baker 2019; Epstein and Kanwisher 1998), and serves broader functions, such as spatial navigation and contextual associations (Aminoff et al. 2013; Bar and Aminoff 2003; Epstein 2008). This functional specialization within the high‐level visual cortex highlights the modular organization of face and scene processing, with the FFA specialized for face recognition and the PPA central to scene‐related tasks.

While early research focused on identifying individual brain regions involved in face and scene perception using functional localizers (Epstein and Kanwisher 1998; Kanwisher et al. 1997), subsequent efforts have shifted toward understanding the connectivity and interactions within distributed neural networks (Baldassano et al. 2016; Brandman and Peelen 2017; Deen et al. 2024; Haxby et al. 2000; Peelen et al. 2009; Pitcher et al. 2011; Rajimehr et al. 2024; Singer et al. 2025; Watson and Andrews 2024). For face perception, Haxby et al. (2000) proposed a foundational distributed model distinguishing between a core system for visual analysis and an extended system for further processing. The core system is comprised of the inferior occipital gyrus for early perception of facial features, the fusiform gyrus (FFA) for representing the invariant aspects of faces that underlie identity, and the superior temporal sulcus (STS) for representing changeable aspects like eye gaze and expression that facilitate social communication. Extended systems then process this information for functions like emotion recognition and directing spatial attention (Haxby et al. 2000).

Similarly, scene perception relies on a distributed cortical network composed of three core regions: the PPA, the occipital place area (OPA), and the retrosplenial complex (RSC) (Epstein and Baker 2019). This network is further organized into two distinct subnetworks (Baldassano et al. 2016; Epstein and Baker 2019). A posterior network, including the OPA and posterior PPA, is strongly connected with visual and dorsal attention networks and is thought to process immediate visual features like spatial layout. In contrast, an anterior network, including the anterior PPA and RSC, connects robustly with the hippocampus, default mode, and frontoparietal control networks to support higher‐level memory and navigational functions (Baldassano et al. 2016; Watson and Andrews 2024). This functional and structural division is believed to support distinct computational goals such that the OPA supports visually guided navigation through the immediate environment, the RSC supports map‐based navigation to out‐of‐sight locations, and the PPA, in a departure from earlier views, primarily supports scene categorization rather than navigation (Dilks et al. 2022). Recent high‐resolution fMRI work further indicates that this scene‐selective network extends into superior parietal cortex, including a scene‐selective region in the posterior intraparietal gyrus that responds selectively to ego‐motion in naturalistic scenes (Kennedy et al. 2024).

Recent research indicates that the brain's intrinsic functional and anatomical connectivity patterns can predict task‐evoked activations and relate to behavioral performance (Bedini et al. 2025; Gomez et al. 2015; Molloy et al. 2024; Saygin et al. 2012; Soyuhos and Baldauf 2023; Zhu et al. 2011). For instance, Saygin et al. (2012) demonstrated that structural connectivity profiles of the fusiform gyrus could predict individual face‐selective responses in the FFA, highlighting a strong link between anatomical connections and functional specialization. Building on this finding, Gomez et al. (2015) identified separate white‐matter pathways associated with face‐ and place‐selective regions and showed that local diffusion properties of these tracts correlated with category‐specific behavioral performance. In addition to these anatomical findings, intrinsic functional connectivity patterns have proven highly informative: Zhu et al. (2011) found that resting‐state functional connectivity strength between the occipital face area and the FFA correlated with individual differences in face recognition abilities. Taken together, these studies suggest that the brain's structural and functional connectivity profiles are closely linked to functional specialization and individual differences in behavior.

Complementary work on mental imagery of familiar people and places has highlighted the broader networks in which face‐ and scene‐selective regions are embedded. fMRI studies have revealed category‐selective subdivisions within medial parietal cortex that are preferentially recruited during recall of people versus places and show distinct resting‐state connectivity with anterior portions of face‐ and scene‐selective ventral temporal cortex (Silson et al. 2019). Extending this view, Steel et al. (2021) identified a network of place‐memory areas immediately anterior to core scene‐perception regions that selectively respond when recalling or viewing familiar locations and interface with hippocampal spatial‐memory systems. More recently, Scrivener et al. (2025) combined EEG with fMRI to characterize the temporal dynamics of this people/place imagery network, demonstrating that category‐selective imagery representations emerge in both medial parietal and ventral temporal cortex and then propagate toward primary visual cortex. Our recent work further showed that mental imagery of faces versus scenes can be decoded from category‐specific patterns of endogenously driven functional connectivity (Mantegna et al. 2025). Overall, these studies indicate that both spontaneous intrinsic activity and imagery‐driven endogenous activity within face‐ and scene‐selective networks carry information about face and scene perception. Understanding how specialized regions like the FFA and PPA are organized within such intrinsic networks is therefore important, as this architecture is thought to provide the scaffold for efficient task‐related processing and may explain individual differences in perceptual abilities (Fox and Raichle 2007; Fox et al. 2005; Mennes et al. 2010; Zou et al. 2013).

In our study, we investigated the resting‐state functional connectivity patterns of the FFA and PPA. Utilizing data from the Human Connectome Project (HCP), we employed both functional magnetic resonance imaging (fMRI) and magnetoencephalography (MEG) to characterize their intrinsic networks. First, using fMRI, we found that the FFA and PPA anchor spatially segregated intrinsic networks whose functional architecture aligns with their known task‐based networks. Second, our MEG analyses revealed that this spatial segregation is reflected in the amplitude coupling of beta and gamma bands and uncovered frequency‐specific directional dynamics, with incoming influences to these core regions carried by higher frequencies and outgoing influences by lower frequencies. Finally, we demonstrated that the strength of this intrinsic architecture is predictive of individual differences in behavior. Specifically, we established a functional double dissociation: connectivity patterns within the FFA network predicted individual performance on a face‐matching task, whereas connectivity within the PPA network predicted performance on scene‐processing tasks, but not vice versa.

## 2 Results

### 2.1 Identification of FFA and PPA Seed Regions

**FIGURE 1:**
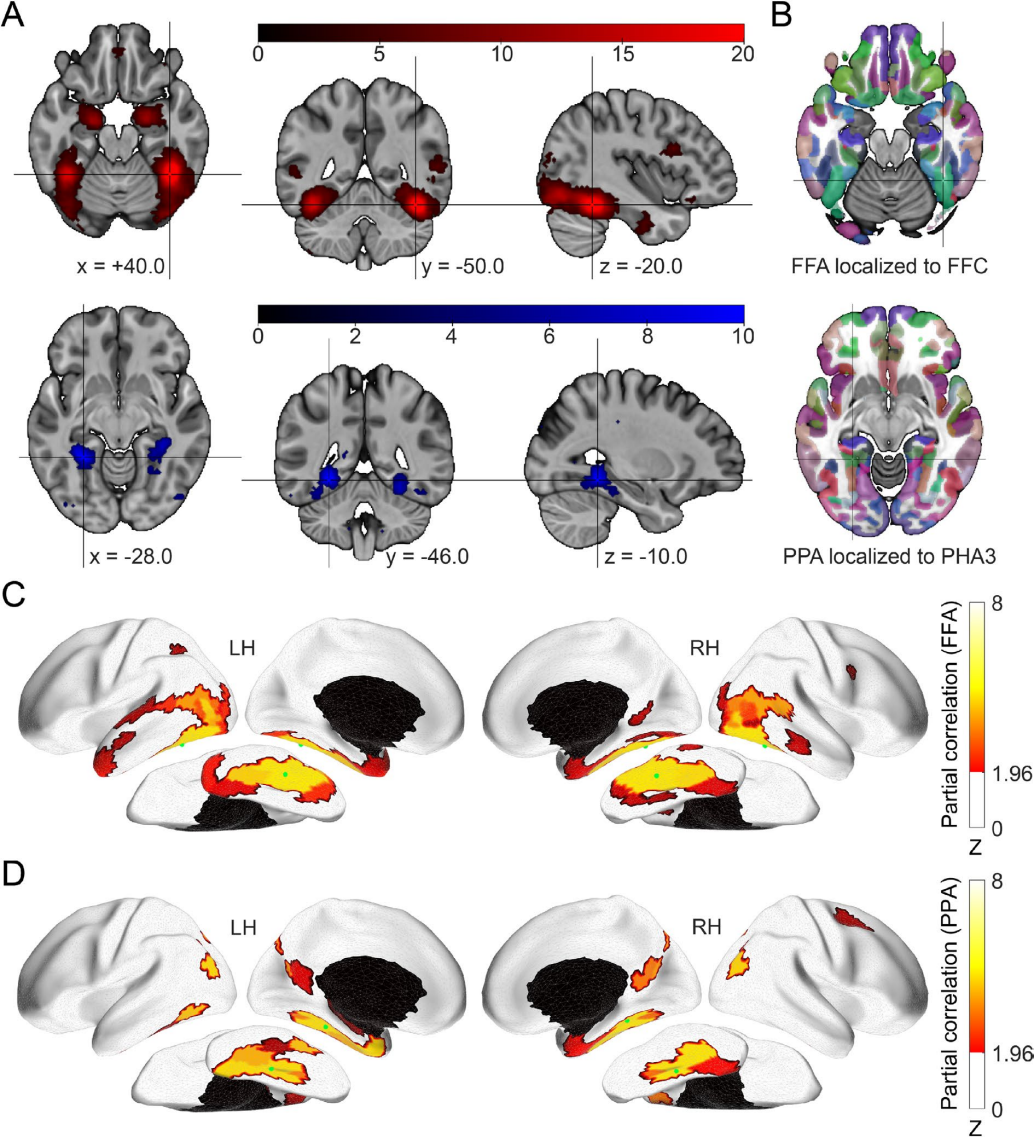
Anatomical locations and fMRI connectivity maps of the Fusiform Face Area (FFA) and Parahippocampal Place Area (PPA). (A) Neurosynth meta‐analytic association maps generated for “face” (top row, red) and “place” (bottom row, blue) queries. For the “face” map, the highlighted peak localizes the FFA to the FFC parcel in the right hemisphere (MNI coordinate in LPI order: +40.0, −50.0, −20.0). For the “place” map, the highlighted peak localizes the PPA to the PHA3 parcel in the left hemisphere (MNI coordinate in LPI order: −28.0, −46.0, −10.0). (B) The location of the parcels in the Human Connectome Project Multi‐Modal Parcellation (HCP‐MMP1) atlas corresponding to the FFA and PPA. (C) Whole‐brain resting‐state fMRI functional connectivity map of the FFA seed region. (D) Whole‐brain resting‐state fMRI functional connectivity map of the PPA seed region. For panels (B) and (C), connectivity is displayed for the left (LH) and right (RH) hemispheres on each ipsilateral side. The green dots mark the seed regions. Statistical significance was assessed using a *p* < 0.05 threshold, FDR‐corrected for 180 ROIs, and represented as *z*‐scores.

We first identified the FFA and PPA seed regions within the multi‐modal parcellation atlas (HCP‐MMP1; Glasser et al. 2016) using a Neurosynth meta‐analysis combined with a clustering approach (Figure 1A and Table S1, see Methods). For the “face” association map, the largest cluster showed peak activation (MNI: +40.0, −50.0, −20.0) located within the FFC parcel, with its center of mass (MNI: +42.3, −59.7, −13.3) situated in the PH parcel, both in the right hemisphere. The second‐largest “face” cluster was localized in the left hemisphere, with both its peak activation (MNI: −40.0, −52.0, −22.0) and center of mass (MNI: −37.8, −65.5, −13.7) positioned within the FFC parcel. For the “place” association map, the largest cluster, with both peak activation (MNI: −28.0, −46.0, −10.0) and center of mass (MNI: −29.7, −45.6, −10.3), was localized within the PHA3 parcel in the left hemisphere. The second‐largest “place” cluster exhibited peak activation (MNI: +28.0, −44.0, −14.0) within the VVC parcel, while its center of mass (MNI: +29.7, −43.0, −11.7) was localized to the PHA3 parcel in the right hemisphere (Table S1). These findings identified the FFC parcel as most frequently associated with face‐related activations, and the PHA3 parcel as most frequently associated with scene‐related activations, leading to their selection as seed regions for the FFA and PPA, respectively.

### 2.2 fMRI Connectivity Reveals Distinct Networks for FFA and PPA

**FIGURE 2:**
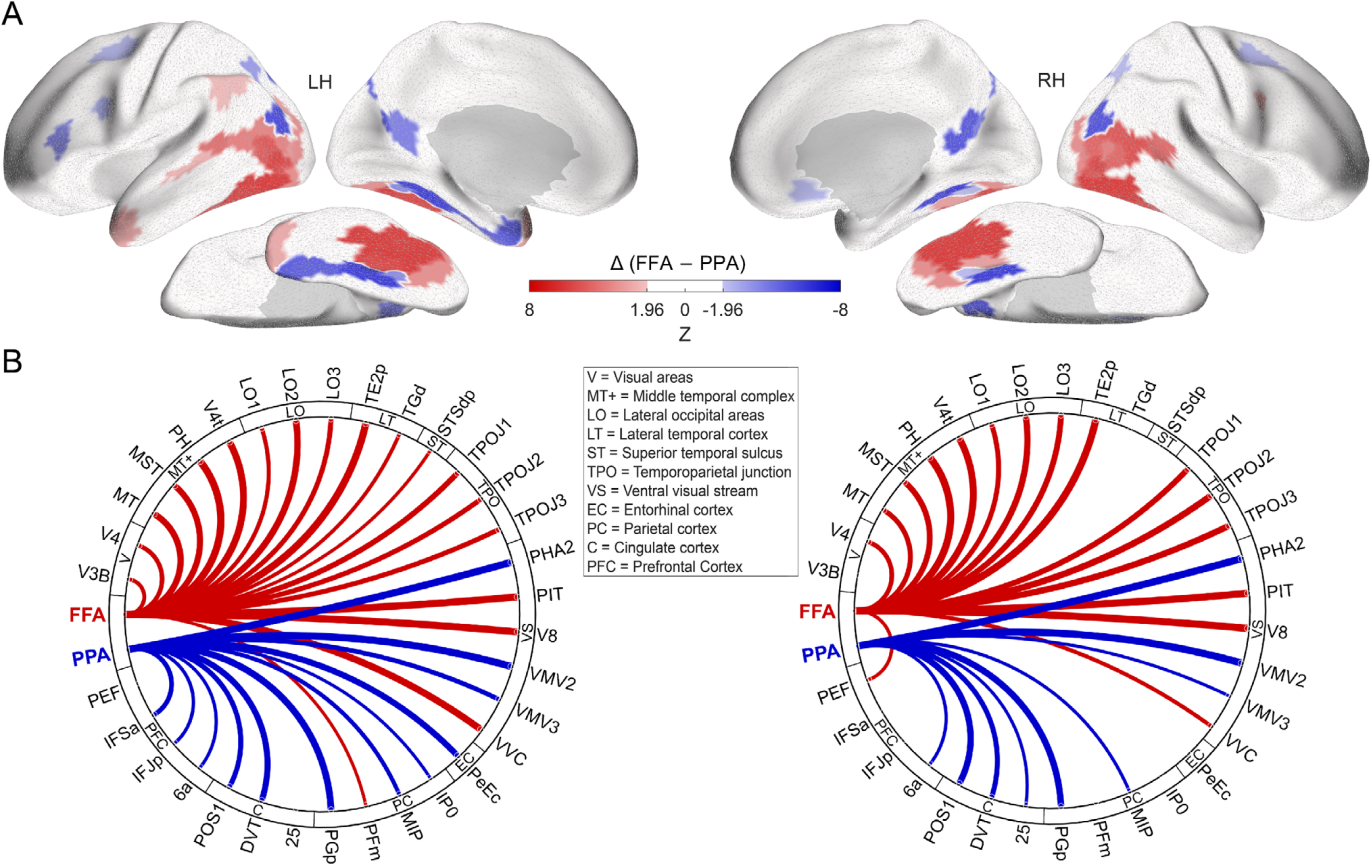
Contrasting fMRI connectivity profiles of the Fusiform Face Area (FFA) and Parahippocampal Place Area (PPA). (A) Surface maps display the direct contrast between the whole‐brain connectivity profiles of FFA and PPA, with red indicating regions showing significantly stronger connectivity with FFA than PPA, and blue indicating regions with stronger connectivity with PPA than FFA. (B) Circular graphs for the left (LH) and right (RH) hemispheres illustrate the predominant functional connections between the FFA and PPA seed regions and the target regions from panel A. Red lines denote preferential connectivity with FFA, and blue lines with PPA; line thickness corresponds to the strength of this preferential connectivity (*z*‐score). Statistical significance for these contrasts was assessed using a *p* < 0.05 threshold, FDR‐corrected for 180 ROIs, with differences represented as *z*‐scores. Abbreviations for the ROI groupings shown in the figure include: C = Cingulate cortex; EC = Entorhinal‐perirhinal cortex; LO = Lateral occipital areas; LT = Lateral temporal cortex; MT+ = Middle temporal complex; PC = Parietal cortex; PFC = Prefrontal Cortex; ST = Superior temporal sulcus; TPO = Temporoparietal junction; V = Visual areas; VS = Ventral visual stream.

Next, we examined the whole‐brain fMRI connectivity patterns associated with these seeds in a sample of 55 HCP participants. The FFA seed exhibited functional connectivity primarily with lateral occipital, ventral temporal, and lateral temporal cortical regions within each hemisphere (Figure 1C; see Table S2 for full list of significant connections). In contrast, the PPA seed showed strong connectivity largely with medial temporal lobe, posterior cingulate cortex, and inferior parietal cortex (Figure 1D; see Table S3 for full list). To identify regions selectively associated with face versus scene processing networks, we directly compared the connectivity profiles of the FFA and PPA seeds in each hemisphere (Wilcoxon signed‐rank test, *p* < 0.05, FDR‐corrected *q* < 0.05). This contrast revealed distinct sets of regions preferentially connected to either the FFA or the PPA (Figure 2). Specifically, 20 parcels demonstrated significantly stronger functional connectivity with the FFA seed region (Table 1). These FFA‐preferential regions included areas within the visual cortex (V3B, V4), middle temporal complex (MT complex: MT, MST, PH, V4t), lateral occipital cortex (LO1‐3), lateral temporal cortex (TE2p), STS (STSdp), temporoparietal junction (TPOJ1‐3), ventral visual stream (PIT, V8, VVC), inferior parietal cortex (PFm), and premotor eye fields (PEF). Conversely, 13 parcels showed significantly stronger functional connectivity with the PPA seed region (Table 1). These PPA‐preferential regions included areas within the ventral medial visual cortex (VMV2‐3), adjacent parahippocampal cortex (PHA2), the entorhinal‐perirhinal cortex (PeEc), medial and inferior parietal cortex (MIP, PGp, IP0), anterior and posterior cingulate cortex (25, DVT, POS1), superior premotor cortex (6a), and inferior frontal cortex (IFJp, IFSa). The specific parcels, their anatomical labels, and the statistics indicating the strength and significance of their preferential connectivity with either the FFA or PPA seed in each hemisphere are detailed in Table 1. These 33 differentially connected regions (20 FFA‐preferential, 13 PPA‐preferential) were subsequently used as target regions of interest (ROIs) for the MEG connectivity analyses.

**TABLE 1:** Brain regions exhibiting predominant functional connectivity with the Fusiform Face Area (FFA) and Parahippocampal Place Area (PPA).

Parcellation	Brain region	Left hemisphere		Right hemisphere	
		*p*	*z*‐score	*p*	*z*‐score
FFA‐network: areas predominantly connected with FFA					
LO1	Lateral Occipital Area	< 0.01	3.18	< 0.001	3.88
LO2	Lateral Occipital Area	< 0.001	5.32	< 0.001	5.08
LO3	Lateral Occipital Area	< 0.001	3.87	< 0.001	4.65
MST	Medial Superior Temporal (MT+ Complex)	< 0.001	4.94	< 0.001	4.62
MT	Middle Temporal (MT+ Complex)	< 0.001	5.09	< 0.001	3.92
PEF	Premotor Eye Field	—	—	< 0.05	2.42
PFm	Inferior Parietal Cortex	< 0.05	2.12	—	—
PH	Area PH (MT+ Complex)	< 0.001	5.91	< 0.001	5.93
PIT	Posterior Inferior Temporal Complex	< 0.001	5.91	< 0.001	5.93
STSdp	Superior Temporal Sulcus	< 0.05	2.29	—	—
TE2p	Lateral Temporal Cortex	< 0.001	5.91	< 0.001	5.93
TGd	Temporal Polar Cortex	< 0.01	2.71	—	—
TPOJ1	Temporo‐Parieto‐Occipital Junction	< 0.001	4.41	< 0.001	4.55
TPOJ2	Temporo‐Parieto‐Occipital Junction	< 0.001	3.92	< 0.001	5.27
TPOJ3	Temporo‐Parieto‐Occipital Junction	< 0.001	3.82	< 0.001	5.10
V3B	Visual Area V3B	< 0.01	2.92	—	—
V4	Visual Area V4	< 0.001	3.47	< 0.001	3.52
V4t	Area V4t (MT+ Complex)	< 0.001	5.61	< 0.001	5.57
V8	Visual Area V8	< 0.001	5.89	< 0.001	5.93
VVC	Ventral Visual Complex	< 0.001	5.61	< 0.01	2.97
PPA‐network: areas predominantly connected with PPA					
25	Subgenual Cingulate Area	—	—	< 0.05	2.11
6a	Superior Premotor Cortex	< 0.05	2.25	< 0.05	2.24
DVT	Posterior Cingulate Cortex	< 0.001	4.86	< 0.001	3.94
IFJp	Inferior Frontal Junction	< 0.05	2.42	—	—
IFSa	Inferior Frontal Sulcus	< 0.001	3.67	—	—
IP0	Inferior Parietal Cortex	< 0.05	2.12	—	—
MIP	Medial Intraparietal Area	< 0.01	3.14	< 0.05	2.19
PGp	Inferior Parietal Cortex	< 0.001	5.46	< 0.001	5.23
PHA2	Parahippocampal Area	< 0.001	5.91	< 0.001	5.93
POS1	Posterior Cingulate Cortex	< 0.001	3.94	< 0.001	5.11
PeEc	Perientorhinal and Ectorhinal Complex	< 0.001	4.92	—	—
VMV2	Ventral Medial Visual Area	< 0.001	5.91	< 0.001	5.93
VMV3	Ventral Medial Visual Area	< 0.001	3.62	< 0.05	2.10

*Note:* This table lists cortical regions from the Human Connectome Project multi‐modal parcellation (HCP‐MMP1) atlas, classifying each region according to its preferential connectivity with FFA or PPA (Figure 2). The “Parcellation” column names the region, while the “Brain Region” column provides an approximate functional label based on Glasser et al. (2013). Separate columns display the statistical significance (*p*‐value) and strength (*z*‐score) of the functional connectivity for the left (LH) and right (RH) hemispheres. Dashes (−) indicate that significant connectivity was not detected in the corresponding hemisphere.

### 2.3 Frequency‐Specific MEG Connectivity Profiles of FFA and PPA

**FIGURE 3:**
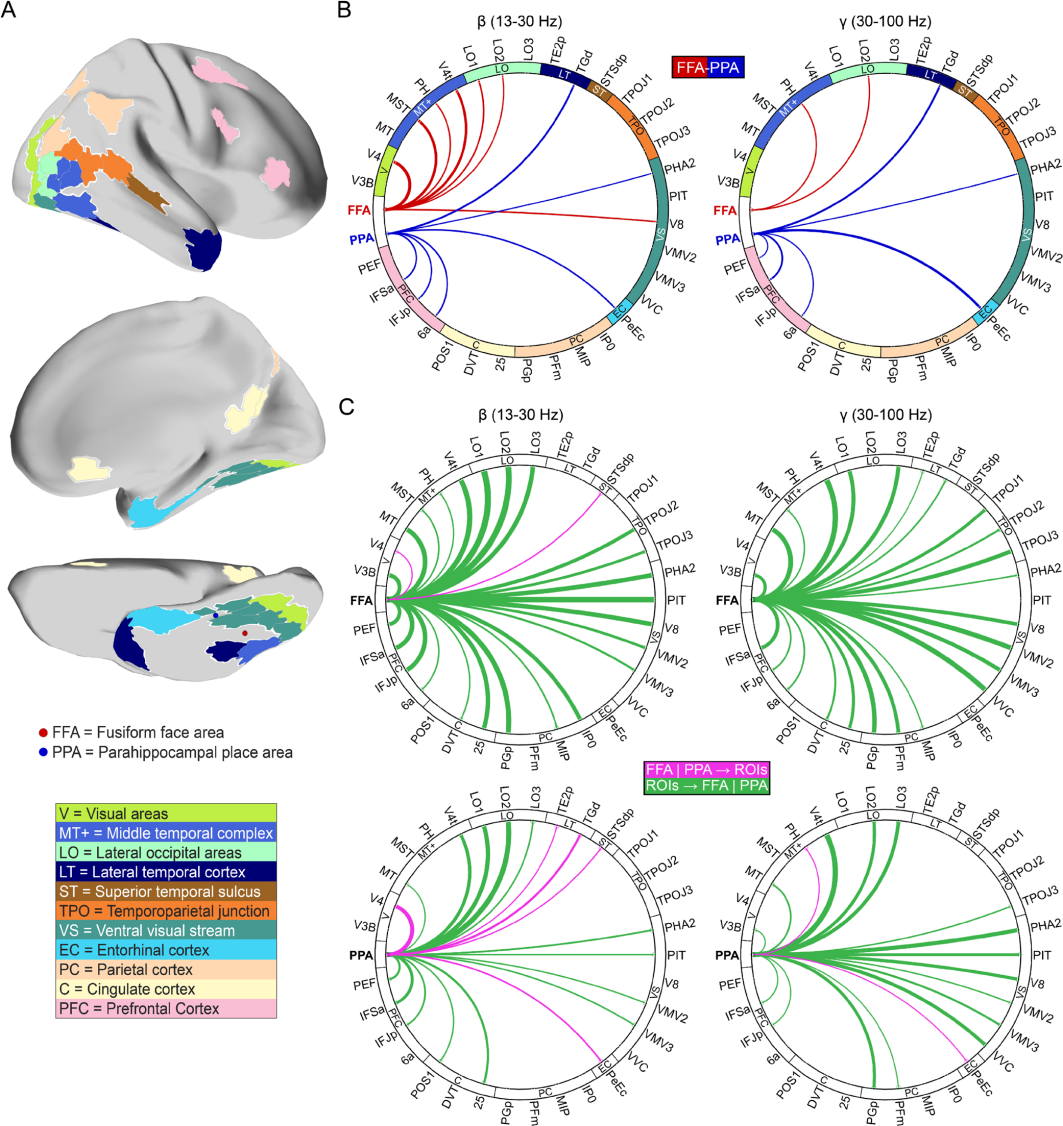
Frequency‐specific connectivity profiles of the Fusiform Face Area (FFA) and Parahippocampal Place Area (PPA). (A) Location of regions of interest (ROIs) from Table 1 on cortex. The parcels with the same color have similar functional labels based on Glasser et al. (2013). (B) Predominant magnetoencephalography (MEG) functional connectivity profiles of the FFA and PPA to the areas shown in panel (A) in the beta (left) and gamma (right) frequency bands across two hemispheres. The results are based on the orthogonalized power envelope correlation method. Red and blue lines indicate preferential connectivity with the FFA and PPA, respectively. There were no significant regions in the other frequency bands. (C) Circular graphs show the directional connectivity in the beta and gamma bands for FFA (top) and PPA (bottom) with the regions in panel (A) across both hemispheres. For both graphs, magenta lines indicate significant outgoing influences (Seed → ROI), while green lines indicate significant incoming influences (ROI → Seed). The results for other frequency bands are in Figure S3. Statistical significance was assessed using a *p* < 0.05 threshold, FDR‐corrected for 33 ROIs, and represented as *z*‐scores. The width of the lines reflects the *z*‐scores for each functional coupling. Abbreviations for the ROI groupings shown in the figure include: C = Cingulate cortex; EC = Entorhinal‐perirhinal cortex; LO = Lateral occipital areas; LT = Lateral temporal cortex; MT+ = Middle temporal complex; PC = Parietal cortex; PFC = Prefrontal Cortex; ST = Superior temporal sulcus; TPO = Temporoparietal junction; V = Visual areas; VS = Ventral visual stream.

To investigate the temporal dynamics and directional interactions within the identified face‐ and place‐preferential networks, we analyzed the MEG data from these same 55 participants, focusing on connectivity between the FFA/PPA seed regions and the 33 target ROIs identified previously via fMRI (Table 1 and Figure 3A). We assessed amplitude and phase coupling using the orthogonalized power envelope correlation and the imaginary part of coherency metrics, respectively (Wilcoxon signed‐rank test, *p* < 0.05, FDR‐corrected *q* < 0.05). Analysis of amplitude coupling in the beta (13–30 Hz) and gamma (30–100 Hz) frequency bands reflected the distinct network segregation observed with fMRI (Figure 3B). In both frequency bands, the FFA seed showed significantly stronger connectivity with target ROIs located primarily in the lateral occipital cortex, MT complex, and earlier visual areas. Conversely, the PPA seed exhibited significantly stronger connectivity with target ROIs in the entorhinal‐perirhinal cortex and the posterior prefrontal cortex in both beta and gamma bands (see Figure S1 for whole‐brain connectivity maps). However, such distinct network segregation with amplitude coupling was not evident in the lower frequency bands; neither the FFA nor PPA seeds demonstrated significant preferential connectivity to these target ROIs in the delta (1–4 Hz), theta (4–8 Hz), or alpha (8–13 Hz) bands. In contrast, phase‐based connectivity analyses only revealed significant preferential connectivity in the beta (13–30 Hz) band, specifically between the PPA and the posterior inferior temporal cortex (PIT) (see Figure S2 for whole‐brain connectivity maps).

We next examined frequency‐specific directional interactions using the partial directed coherence (PDC) method across two hemispheres (Wilcoxon signed‐rank test, *p* < 0.05, FDR‐corrected *q* < 0.05) (Figures 3C and S3). Incoming influences (ROI → Seed), directed toward the FFA and PPA seeds, were predominant in the higher frequencies (beta and gamma) (green lines, Figure 3C). Prominent sources driving these high‐frequency incoming influences included the lateral occipital cortex (LO1‐3), MT complex (MT, V4t), ventral visual stream areas (PHA2, PIT, V8, VMV2‐3, VVC), temporoparietal junction (TPOJ: TPOJ2‐3), and PFC regions (PEF, IFSa, IFJp, 6a). Notably, while contributing significantly to these high‐frequency interactions, influences originating from PFC specifically exhibited their peak strength in the low frequency band. Conversely, outgoing influences (Seed → ROI), originating from the FFA and PPA seeds, were relatively stronger in the lower frequencies (delta, theta, and alpha) (magenta lines, Figure S3). Significant outgoing influences were directed toward target ROIs including area V4, STS (STSdp), TPJ (TPOJ1), ventral visual cortex (VVC), the entorhinal‐perirhinal cortex (PeEc), and posterior cingulate cortex (DVT, POS1). Overall, incoming influences were most robust in the gamma band, while outgoing influences were most robust in the alpha band.

In summary, MEG analyses further characterized the spatial segregation of FFA and PPA networks observed in fMRI, particularly within beta and gamma band amplitude coupling. Additionally, these analyses revealed frequency‐specific temporal dynamics characterized by predominant incoming influences in high frequency bands (beta, gamma) and relatively stronger outgoing influences in low frequency bands (delta, theta, and alpha).

### 2.4 Intrinsic Network Connectivity Predicts Behavioral Performance

**FIGURE 4:**
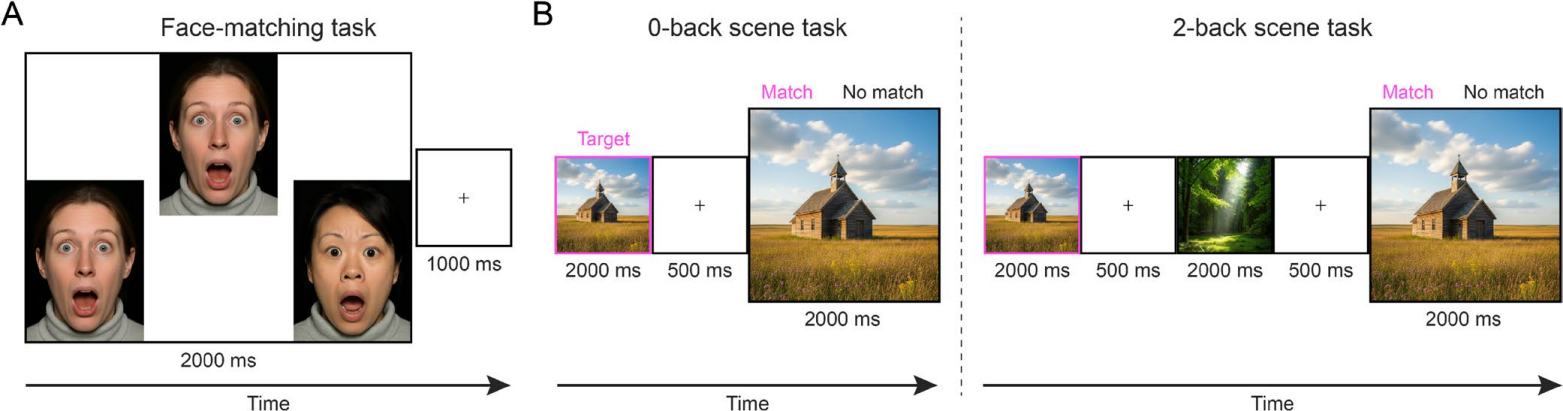
Face‐matching and *n*‐back scene paradigms. (A) In the face‐matching task, participants identified which of the two choice faces at the bottom matched the target face at the top. Each trial consisted of a 2000 ms stimulus presentation followed by a 1000 ms fixation interval. (B) In the scene *n*‐back task, participants performed either a 0‐back (left) or 2‐back (right) working memory task. For both conditions, each stimulus was presented for 2000 ms followed by a 500 ms fixation interval. The illustrative images shown here were computer‐generated.

Finally, using a larger cohort of HCP participants with resting‐state fMRI and behavioral data (see Methods), we investigated whether the distinct intrinsic functional connectivity patterns of the FFA and PPA networks were related to individual differences in performance on the face‐matching and scene *n*‐back tasks (Figure 4). Behavioral performance was characterized by high accuracy and substantial inter‐individual variability in median RT (Figure S4). Mean accuracies were 98.45% (*N* = 350, SD = 3.81%) for the face‐matching task, 94.39% (*N* = 352, SD = 9.14%) for the 0‐back scene task, and 90.22% (*N* = 352, SD = 9.68%) for the 2‐back scene task. The corresponding mean median RTs were 784.43 ms (*N* = 350, SD = 130.87 ms), 723.45 ms (*N* = 352, SD = 140.36 ms), and 920.78 ms (*N* = 352, SD = 170.49 ms), respectively. Spearman correlations between median RT and accuracy were 0.01 (*p* = 0.86) for face‐matching, −0.36 (*p* < 0.001) for the 0‐back scene task, and −0.10 (*p* = 0.07) for the 2‐back scene task, indicating that RT variability was not primarily driven by response strategy and instead reflected true performance differences.

We employed connectome‐based predictive modeling (CPM) using fMRI partial correlations within the previously defined FFA‐preferential network (37 regions including bilateral seeds and significant ROIs from Table 1) and PPA‐preferential network (23 regions including bilateral seeds and significant ROIs from Table 1). These networks were used to predict median RTs from the face‐matching task and the 0‐back and 2‐back scene tasks, respectively (task details in Figure 4). All models were rigorously tested using leave‐one‐out cross‐validation and permutation testing (1000 iterations) for statistical significance.

**FIGURE 5:**
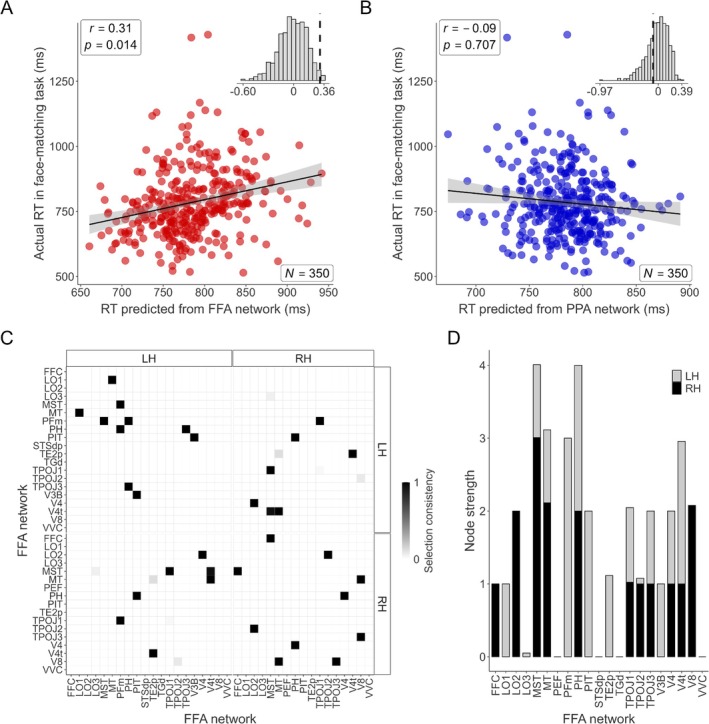
Connectome‐based predictive modeling (CPM) of median reaction times in the face‐matching task. (A) The scatter plot compares actual median reaction times (RT; ms) with those predicted by CPM, based on resting‐state fMRI connectivity within the FFA network (37 regions, including bilateral seeds and significant ROIs from Table 1). The black line represents the linear regression fit, and the gray shading indicates the 95% confidence interval. The inset histogram displays the null distribution of correlations obtained through permutation testing, with the dashed vertical line marking the observed correlation. (B) The same procedure as in (A) but using a CPM model based on connectivity within the PPA network (23 regions, including bilateral seeds and significant ROIs from Table 1). (C) The adjacency matrix visualizes the connectivity pairs driving the successful prediction shown in (A). The shading of each cell represents the proportion of cross‐validation folds in which that edge was selected as a significant predictor of reaction time (selection consistency). (D) The bar chart displays node strength, a measure of each region's total contribution to the predictive model, calculated by summing the selection consistency scores in (C) across all connections involving that region.

First, we tested if connectivity within the FFA network could predict performance on the face‐matching task (*N* = 350 participants). The CPM analysis revealed a significant positive correlation between predicted and actual median RTs (Spearman's *r* = 0.31, 95% CI [0.22, 0.40], df = 348, *p* = 0.014, permutation test; Figure 5A), indicating that stronger intrinsic connectivity patterns within this network were associated with faster face‐matching performance. In contrast, a control model using connectivity within the PPA network failed to predict face‐matching RTs (Spearman's *r* = −0.09, 95% CI [−0.19, 0.02], df = 348, *p* = 0.707, permutation test; Figure 5B), demonstrating the specificity of the FFA network's relationship with face processing behavior. The successful prediction was driven by a distributed set of connections consistently selected as predictive features across subjects (Figure 5C), with regions within the MT complex (MST, MT, PH, V4t) and inferior parietal cortex (PFm) showing the highest number of predictive pairs within the FFA network.

**FIGURE 6:**
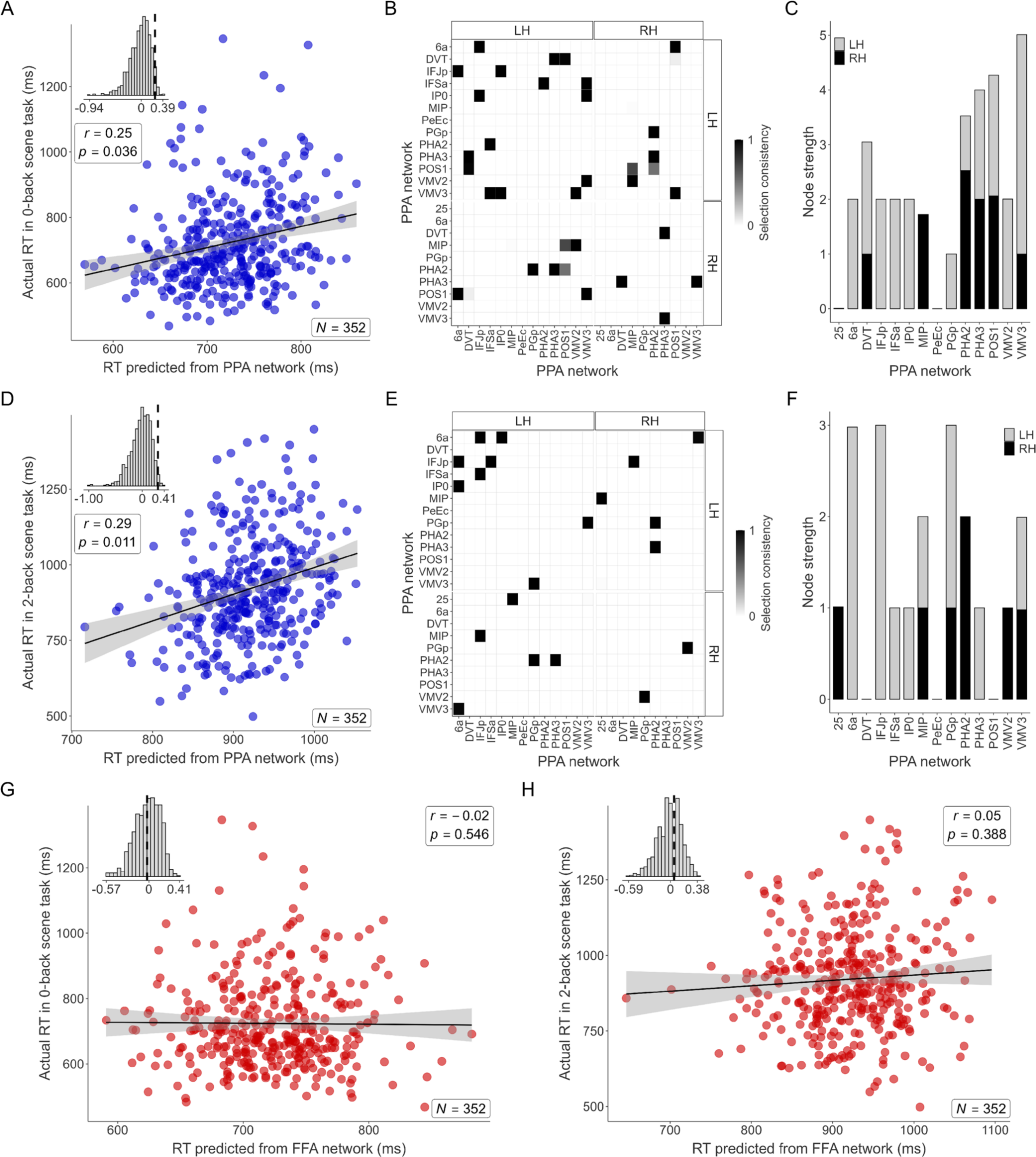
Connectome‐based predictive modeling (CPM) of median reaction times in 0‐back and 2‐back scene tasks. (A) The scatter plot compares actual median reaction times (RTs; ms) from the 0‐back scene task with those predicted by CPM, based on resting‐state fMRI connectivity within the PPA network (23 regions, including bilateral seeds and significant ROIs from Table 1). The black line represents the linear regression fit, and the gray shading indicates the 95% confidence interval. The inset histogram displays the null distribution of correlations obtained through permutation testing, with the dashed vertical line marking the observed correlation. (B) The adjacency matrix visualizes the connectivity pairs driving the successful prediction shown in (A). The shading of each cell represents the proportion of cross‐validation folds in which that edge was selected as a significant predictor of reaction time (selection consistency). (C) The bar chart displays node strength, a measure of each region's total contribution to the predictive model, calculated by summing the selection consistency scores in (B) across all connections involving that region. (D–F) Corresponding results for the 2‐back scene task using the same PPA network as in (A–C), showing the prediction scatter plot (D), adjacency matrix (E), and node strength (F). (G) The same procedure as in (A) but predicting the 0‐back scene task RTs using a CPM model based on connectivity within the FFA network (37 regions, including bilateral seeds and significant ROIs from Table 1). (H) Similar to (G), but predicting the 2‐back scene task RTs using the FFA network.

Next, we assessed the relationship between PPA network connectivity and performance on the scene *n*‐back tasks (*N* = 352 participants). Connectivity within the PPA network significantly predicted median RTs for the 0‐back scene task (Spearman's *r* = 0.25, 95% CI [0.14, 0.35], df = 350, *p* = 0.036, permutation test; Figure 6A). The control model using the FFA network connectivity did not significantly predict 0‐back RTs (Spearman's *r* = −0.02, 95% CI [−0.13, 0.08], df = 350, *p* = 0.546, permutation test; Figure 6E), further supporting network specificity. Similarly, connectivity within the PPA network also significantly predicted median RTs for the more demanding 2‐back scene task (Spearman's *r* = 0.29, 95% CI [0.20, 0.39], df = 350, *p* = 0.011, permutation test; Figure 6C). The corresponding control model using FFA network connectivity failed to predict 2‐back RTs (Spearman's *r* = 0.05, 95% CI [−0.06, 0.16], df = 350, *p* = 0.388, permutation test; Figure 6F). For both the 0‐back and 2‐back scene tasks, the successful predictions using the PPA network were based on distributed patterns of connectivity (Figure 6B,D). Notably, the specific patterns differed: the 0‐back prediction drew heavily on connections within parahippocampal (PHA2, PHA3), posterior cingulate (POS1, DVT), and ventral medial visual (VMV3) cortex (Figure 6B), while the 2‐back prediction showed increased reliance on connections involving frontal (6a, IFJp) and parietal (PGp, MIP) regions (Figure 6D).

To assess whether these predictive relationships were driven specifically by connections involving the FFA and PPA seeds, we performed a control CPM analysis in which all edges connected to the seed regions were removed from each network (Figure S5). The seed‐excluded FFA network continued to predict median RTs in the face‐matching task (Spearman's *r* = 0.30, 95% CI [0.21, 0.40], *p* = 0.014, permutation test; *N* = 350). Likewise, the seed‐excluded PPA network continued to predict median RTs in the 2‐back scene task (Spearman's *r* = 0.27, 95% CI [0.17, 0.36], *p* = 0.016; *N* = 352), whereas prediction for the 0‐back scene task was weaker and no longer significant (Spearman's *r* = 0.18, 95% CI [0.07, 0.28], *p* = 0.111; *N* = 352). These findings indicate that behaviorally relevant information is distributed across the intrinsic networks anchored by FFA and PPA, while connections involving the seed regions still make an important contribution, particularly for the 0‐back scene condition.

Taken together, these CPM results demonstrate a double dissociation: intrinsic resting‐state functional connectivity within the FFA network specifically predicted individual differences in face‐matching performance, whereas connectivity within the PPA network specifically predicted performance on both 0‐back and 2‐back scene tasks, highlighting a significant link between the brain's intrinsic network architecture and domain‐specific perceptual processing capabilities.

### 2.5 Posterior Versus Anterior PPA Networks

In our main analyses, the PPA seed was defined from a Neurosynth “place” association map, which localized to the PHA3 parcel of the HCP‐MMP1 atlas (Table S1). Based on the y ≈−42 mm boundary proposed by Baldassano et al. (2016), PHA3 falls in posterior PPA, whereas the neighboring PHA2 parcel lies in a more anterior PPA territory. To test whether our behavioral effects depended specifically on a posterior versus anterior PPA seed, we repeated the connectivity and CPM analyses using PHA2 as an alternative, more anterior PPA seed while keeping the FFA seed (FFC) unchanged. The resulting FFA‐preferential network closely resembled the original face network, including bilateral lateral occipital cortex (LO1–3), MT complex (MT, MST, FST, V4t, PH), ventral occipitotemporal regions (V3B, V4, V8, VVC, PIT, TF, TGd), superior temporal sulcus (STSdp), temporoparietal junction areas (TPOJ1–3), and inferior parietal cortex (PFm), as well as frontal and parietal nodes such as PEF, LIPd, and 8BL. In contrast, the PHA2‐anchored PPA network comprised regions more strongly weighted toward medial temporal, medial parietal, and cingulate cortex, including bilateral PHA1–3, PGp/PGs, POS1, 31a, and 7Pm, together with frontal and insular regions such as 8Ad and AAIC (Wilcoxon signed‐rank test, *p* < 0.05, FDR‐corrected *q* < 0.05).

We next tested the behavioral relevance of this anterior PPA–anchored network using the same CPM framework as in the main analyses. Whereas the posterior PPA (PHA3) network robustly predicted median RTs in both the 0‐back and 2‐back scene tasks (Figure 6), the anterior PPA (PHA2) network did not. For the 0‐back task, the correlation between predicted and observed median RTs was small and non‐significant (Spearman's *r* = 0.07, 95% CI [−0.03, 0.17], *p* = 0.35). For the 2‐back task, the PHA2‐anchored CPM failed to predict RTs, showing a negative cross‐validated correlation between predicted and observed values (Spearman's *r* = −0.31; permutation *p* = 0.92). In contrast, the FFA‐anchored network continued to predict median RTs in the face‐matching task with similar strength when the PHA2 seed was used in place of PHA3 (Spearman's *r* = 0.30, 95% CI [0.20, 0.40], *p* = 0.018; permutation test). Together, these posterior–anterior control analyses indicate that, for the scene *n*‐back tasks used here, behaviorally relevant intrinsic connectivity is specifically anchored in a posterior PPA region, whereas connectivity within a more anterior PPA network does not significantly predict task performance.

## 3 Discussion

This study provides compelling evidence that the core regions for face (FFA) and scene (PPA) perception are embedded within distinct intrinsic functional networks. These networks are characterized by unique spatial and temporal connectivity profiles that are present even during task‐free rest. Leveraging the complementary strengths of fMRI and MEG, we demonstrated three core findings: First, fMRI revealed spatially segregated networks primarily characterized by the FFA's preferential connectivity with lateral occipitotemporal, inferior temporal, and temporoparietal regions versus the PPA's connectivity with ventral medial visual, posterior cingulate, and entorhinal‐perirhinal areas. Second, MEG analyses showed that this spatial segregation is mirrored in the amplitude coupling of higher frequency bands (beta, gamma), and further uncovered frequency‐specific directional dynamics, highlighting dominant incoming influences (ROI → Seed) in higher frequencies and relatively stronger outgoing influences (Seed → ROI) in lower frequencies. Third, our predictive model established a direct behavioral relevance for this intrinsic architecture: connectivity patterns within the FFA network specifically predicted individual face‐matching performance, whereas connectivity within the PPA network specifically predicted performance on both 0‐ and 2‐back scene tasks, and not vice versa, demonstrating a clear double dissociation.

### 3.1 Spatial Organization of FFA and PPA Networks

The distinct fMRI connectivity profiles observed here strongly reinforce the principle that the functional specialization of the FFA and PPA (Epstein and Kanwisher 1998; Kanwisher et al. 1997) is embedded within the brain's intrinsic functional architecture, resonating with extensive work showing correspondence between resting‐state and task‐based networks (Cole et al. 2014; Fox and Raichle 2007; Smith et al. 2009; Tavor et al. 2016) and confirming this intrinsic organization provides a scaffold for efficient perceptual processing. Specifically, the FFA's preferential connectivity with the lateral occipital cortex, MT complex, STS, and TPJ aligns well with established core and extended face processing networks (de Vries and Baldauf 2019; Duchaine and Yovel 2015; Haxby et al. 2000; Müller et al. 2018; Rajimehr et al. 2009, 2024; Von Der Heide et al. 2013). Previous models proposed a division between the FFA for invariant identity processing and the posterior STS (pSTS) for changeable aspects like expression (de Vries and Baldauf 2019; Gobbini and Haxby 2007; Haxby et al. 2000), with extensions highlighting pSTS's role in dynamic identity cues (O'Toole et al. 2002). However, a more recent perspective emphasizes a primary functional division between form processing via the ventral stream and motion processing via the dorsal stream (Bernstein and Yovel 2015). Our connectivity results fit well within this evolving understanding: the FFA's connection with the lateral occipital cortex underscores their role as a ventral pathway analyzing facial form; connectivity with the MT complex might reflect interaction with general motion analysis that interfaces with the specialized processing of facial motion (like expression or dynamic identity) in the STS regions; and the linkage to the TPJ could represent the integration of identity perception with the retrieval of person knowledge and theory of mind processes (Gobbini and Haxby 2007). This revised view, emphasizing form versus motion, also accommodates findings that the FFA processes expression (likely its form components) and the pSTS responds to static cues implying motion (Bernstein and Yovel 2015), highlighting the integrated nature of face perception supported by the observed intrinsic connectivity network.

Conversely, the PPA network's preferential connectivity with adjacent parahippocampal cortex, inferior parietal areas, and posterior cingulate cortex maps onto established networks for scene recognition, spatial navigation, and contextual memory integration (Aminoff et al. 2013; Bar and Aminoff 2003; Caspers et al. 2013; Epstein 2008; Epstein and Baker 2019; Rajimehr et al. 2024). Of particular significance in our results is the strong coupling observed between the PPA and the entorhinal‐perirhinal cortex. This connection likely reflects the critical interplay between scene‐level processing (Epstein and Baker 2019) and the detailed object processing functions attributed to the entorhinal‐perirhinal cortex, especially the perirhinal cortex (Brown and Aggleton 2001; Deshmukh et al. 2012; Murray and Bussey 1999; Suzuki and Naya 2014). The perirhinal cortex is crucial for representing object features, identifying objects (Murray and Bussey 1999), and signaling item familiarity (Brown and Aggleton 2001; Suzuki and Naya 2014). Furthermore, the perirhinal cortex supports various forms of associative memory, linking items together or associating items with rewards or context (Murray and Bussey 1999; Suzuki and Naya 2014). Notably, the lateral entorhinal cortex receives input from the perirhinal cortex (Suzuki and Naya 2014) and represents spatial information derived from landmarks when objects are present, suggesting an integration of object and spatial context (Deshmukh et al. 2012). Therefore, the strong functional connectivity between PPA and the entorhinal‐perirhinal cortex observed here likely facilitates the integration of object identity and familiarity information (Brown and Aggleton 2001; Deshmukh et al. 2012; Murray and Bussey 1999; Suzuki and Naya 2014) with the broader spatial layout and context represented by PPA (Epstein and Baker 2019). This interaction might be essential for building holistic scene representations, enabling functions such as recognizing objects within their typical scenes, associating objects with specific locations (Brown and Aggleton 2001), and potentially using familiar objects as landmarks (Epstein and Baker 2019) within a scene context processed by PPA.

### 3.2 Frequency‐Specific MEG Dynamics

The MEG analyses provided further insights into the temporal and directional dynamics governing communication within these intrinsic networks. First, we found that amplitude coupling in the beta (13–30 Hz) and gamma (30–100 Hz) frequency bands mirrored some important aspects of the spatial segregation observed with fMRI. This reinforces the role of these faster oscillations in maintaining information processing within these specialized resting‐state networks, potentially binding distributed neural populations (de Pasquale et al. 2010; Engel et al. 2001; Hipp et al. 2012; Siegel et al. 2012). Other frequency bands, such as alpha, theta, and delta, did not show such a spatial congruence with the fMRI results. These frequency‐specific effects are consistent with evidence that fMRI BOLD fluctuations are preferentially related to higher‐frequency neuronal population activity: invasive recordings in monkeys and humans have shown that BOLD responses covary most strongly with local field potential power in the gamma range and show opposite relationships for low‐frequency power (Logothetis et al. 2001; Mukamel et al. 2005). Extending this to non‐invasive measures, MEG and EEG studies have demonstrated that resting‐state networks resembling canonical fMRI‐derived RSNs are most clearly expressed in the amplitude envelopes of higher‐frequency oscillations, particularly in the alpha–beta range, and that different networks have characteristic spectral fingerprints across alpha, beta, and gamma bands (Brookes et al. 2011; de Pasquale et al. 2010; Hipp et al. 2012; Mantini et al. 2007; O'Neill et al. 2015). In this context, our finding that FFA‐ and PPA‐anchored networks are selectively expressed in beta‐ and gamma‐band amplitude coupling, but not in lower‐frequency bands, suggests that these higher‐frequency envelopes provide an electrophysiological counterpart of the fMRI seed‐based connectivity for these category‐selective regions.

Compared with amplitude coupling, phase‐based connectivity analyses using the imaginary part of coherency (Figure S2) revealed much sparser patterns of significant coupling for both FFA and PPA. This difference between amplitude‐ and phase‐based metrics is expected. Orthogonalized amplitude‐envelope correlations capture slow co‐modulations of oscillatory power by averaging power over longer time windows, making them relatively robust to noise and small timing jitter. As a result, they typically yield more extensive large‐scale networks (Colclough et al. 2016; Hipp et al. 2012; Siems and Siegel 2020; Soyuhos and Baldauf 2023). By contrast, phase‐coupling methods such as the imaginary part of coherency detect only consistent non‐zero‐lag phase relationships at the carrier frequency, making them more conservative and more sensitive to small desynchronizations. Consequently, even minor noise or timing differences can reduce the apparent connectivity and lead to more focal networks (Colclough et al. 2016; Siems and Siegel 2020). We therefore interpret the phase‐based results as highlighting a more restricted subset of tightly phase‐locked interactions embedded within the broader amplitude‐defined networks.

Notably, the whole‐brain MEG amplitude‐coupling maps (Figure S1) extend further into parietal and frontal cortex than the corresponding fMRI connectivity maps (Figure 1). fMRI and MEG are fundamentally different types of data that are recorded in different ways. MEG measures fast electromagnetic activity relatively directly, whereas fMRI indexes neural activity indirectly via a delayed hemodynamic response reflecting metabolic demands. Some divergence between connectivity patterns derived from these two modalities is therefore likely, and we interpret the differences between them in light of their distinct biophysical and methodological properties. In addition, the connectivity metrics we use in each modality also influence the observed patterns. In the fMRI analysis, we estimate parcel‐wise partial correlations between the seed (FFA or PPA) and each parcel after regressing out the time series of all other parcels (Smith et al. 2013), which attenuates variance shared via indirect pathways and emphasizes regions that uniquely covary with FFA/PPA, mainly within occipito‐temporal cortex and closely related association areas. In contrast, the MEG maps are based on pairwise power‐envelope correlations (Colclough et al. 2016; Hipp et al. 2012), which capture both more specific seed–target interactions and more distributed co‐fluctuations that can involve parietal and frontal hubs. In this sense, the MEG amplitude‐coupling networks show how the FFA‐ and PPA‐centered networks identified in fMRI are embedded within more extended higher‐order fronto‐parietal systems. Together with the frequency‐specific results above, this suggests that beta/gamma amplitude coupling provides an electrophysiological counterpart of the fMRI seed‐based connectivity for FFA and PPA, while the broader whole‐brain amplitude networks may additionally reflect interactions with control and association regions.

### 3.3 Directional Connectivity of FFA and PPA

The directionality analysis revealed frequency‐specific directed interactions between the FFA/PPA seeds and their associated ROIs. Importantly, interpreting these directed influences requires considering the specific anatomical pathways involved, as FFA and PPA regions function as mid‐level hubs in the functional hierarchy. For example, the signals arriving at these seeds (ROI → Seed) can represent feedforward input if originating from lower and parallel visual areas (e.g., V4t, LO1‐3) or feedback if descending from higher cortical regions (e.g., from PFC, TPJ). Similarly, the signals departing from these seeds (Seed → ROI) can represent feedback (e.g., to V4) or feedforward projections (e.g., to STS, entorhinal‐perirhinal cortex). Within this anatomical context, our findings indicated a general principle: incoming influences were predominantly carried by higher frequencies (beta and gamma), integrating signals from diverse cortical sources. In contrast, outgoing influences were relatively stronger in lower frequencies (delta, theta, and alpha), projecting toward both lower and higher processing stages.

These seed‐relative directional effects differ from classical notions of feedforward and feedback signaling, where ascending influences are most prominent in the gamma and theta bands and descending influences are stronger in the alpha–beta range (Bastos et al. 2015; Michalareas et al. 2016). However, the robustness and generality of this mapping are still under discussion, and recent work has emphasized that gamma‐ and alpha/beta‐band activity are better understood as reflecting partially distinct frequency‐specific networks with area‐dependent spectral profiles rather than fixed feedforward and feedback channels (Vinck et al. 2025). Hillebrand et al. (2016) similarly reported that large‐scale resting‐state interactions can form frequency‐dependent reentry loops, with posterior‐to‐anterior flow in the alpha/beta range and anterior‐to‐posterior flow in theta. Because FFA and PPA occupy a mid‐ to high‐level position and are coupled both to earlier visual cortex and to parietal, medial temporal, and prefrontal regions, the “incoming” and “outgoing” influences captured by our directionality analysis at rest are likely to combine both anatomically feedforward and feedback pathways. We therefore interpret the observed pattern of higher‐frequency influences converging on FFA/PPA and lower‐frequency influences originating from them primarily as a frequency‐specific asymmetry in their resting‐state network interactions, rather than as a direct readout of feedforward versus feedback processing.

Taken together, this frequency‐specific directional architecture may form the intrinsic basis for the temporal dynamics observed during active perception. For instance, MEG decoding has shown that the brain begins to process face‐specific information exceptionally early around 61 ms (Cichy et al. 2014). Notably, this process unfolds in a coarse‐to‐fine sequence: the brain extracts categorical information like gender starting around 72 ms, before recognizing a face's specific identity at 91 ms (Dobs et al. 2019). In contrast, feedback‐driven contextual processes, such as a scene facilitating the perception of an object, occur significantly later around 320 ms (Brandman and Peelen 2017). Further investigation is needed to understand how the frequency‐specific directed interactions we observed during rest map onto these task‐evoked temporal dynamics.

### 3.4 Intrinsic Connectivity and Behavioral Performance

Finally, we showed that this intrinsic functional architecture, measured during task‐free rest, is directly and specifically linked to individual cognitive abilities. The CPM results provide robust, cross‐validated evidence for this link, successfully predicting individual differences in face‐matching and scene *n*‐back RTs from fMRI connectivity patterns within the functionally relevant networks. Crucially, the observed double dissociation—the FFA network predicting only face task performance and the PPA network predicting only scene task performance—offers strong evidence that functional specificity is encoded within these intrinsic networks. This work significantly extends previous correlational studies (Zhu et al. 2011) by employing a rigorous predictive framework (Finn et al. 2015; Rosenberg and Finn 2022; Shen et al. 2017) and demonstrating this specificity across both face and scene domains within the same participants. Moreover, the distinct connectivity patterns within the PPA network predicting performance of the 0‐back versus the more demanding 2‐back scene task suggest that subtle variations in intrinsic network organization are sensitive to, and predictive of, the capacity to engage network resources differently based on cognitive load. The increased reliance on connectivity involving frontal (6a, IFJp) and parietal (PGp, MIP) regions for predicting 2‐back performance likely reflects the intrinsic substrate supporting the greater recruitment of working memory and executive control needed for this higher‐load condition (Baldauf and Desimone 2014; Mantegna et al. 2025). This frontoparietal involvement may also support the implementation of top‐down search strategies that create a category‐specific bias for task‐relevant objects in visual cortex (Peelen et al. 2009), with parietal regions like the intraparietal sulcus thought to be a source of these contextual guidance signals (Peelen 2025). Consistent with this network‐level interpretation, a control analysis using seed‐excluded versions of the face and scene networks (Figure S5) showed that connectivity among non‐seed regions alone remained sufficient to predict face‐matching and 2‐back scene performance, indicating that the behaviorally relevant information is distributed across networks anchored by FFA and PPA rather than confined to the seed regions themselves.

### 3.5 Posterior–Anterior Organization of PPA

Previous work has shown that PPA contains a posterior subdivision that is more closely coupled to occipital and OPA regions and supports visually driven analysis of scene layout, and a more anterior subdivision that shows stronger connectivity with medial parietal, parietal, and hippocampal areas and is thought to be more involved in contextual, memory‐ and navigation‐related processing of places (Baldassano et al. 2016, 2013; Silson et al. 2016). In the HCP‐MMP1 atlas, our Neurosynth‐derived PPA seed falls in the PHA3 parcel at MNI coordinates that are posterior to the previously reported boundary between posterior and anterior PPA. In complementary analyses, we used the adjacent PHA2 parcel as a more anterior PPA seed and showed that this seed anchors a network more strongly weighted toward medial temporal, medial parietal, and cingulate cortex, consistent with prior descriptions of an anterior, context‐ and memory‐related scene network.

Importantly, only the posterior PPA (PHA3) network predicted individual differences in performance on the 0‐back and 2‐back scene tasks, whereas the more anterior PPA (PHA2) network did not show significant predictive power. For the specific scene *n*‐back paradigms used in the HCP protocol, this pattern suggests that the behaviorally relevant intrinsic architecture is anchored in a posterior PPA region and its associated visual/contextual network, whereas anterior PPA and its broader mnemonic/navigation network may be more strongly engaged by tasks that require explicit recall of familiar environments or goal‐directed navigation. We did not further subdivide FFA along a posterior–anterior axis, as an analogous gradient has been less clearly established for this region, and our FFA seed (FFC) corresponds closely to canonical fusiform face‐selective cortex.

### 3.6 Limitations and Future Directions

Importantly, our findings stem from resting‐state data, and directly comparing these intrinsic patterns with connectivity dynamics during active face and scene perception using both fMRI and MEG is a crucial next step to fully understand task‐dependent network modulation. Additionally, assessing the generalizability of these findings beyond the healthy young adult HCP sample requires investigating these network profiles across diverse ages and in clinical populations with known perceptual differences (e.g., prosopagnosia, autism spectrum disorder). Such research would allow exploration of potential interactions across domains (face/scene) and provide further tests of the functional specificity and dissociation of the underlying FFA and PPA networks. Addressing these points, alongside exploring more advanced analyses like dynamic functional connectivity or cross‐frequency coupling, promises to significantly enhance our understanding of how these specialized networks dynamically support face and scene perception.

In conclusion, our results show that the FFA and PPA anchor distinct intrinsic functional networks, characterized not only by unique spatial topographies but also by specific temporal and directional dynamics observable in frequency‐specific MEG signals. Critically, we established that this intrinsic network architecture, present during task‐free rest, is functional as connectivity patterns within these networks specifically predict individual differences in face and scene processing abilities. These findings underscore the power of integrating multimodal neuroimaging to reveal the brain's specialized functional organization and understand the fundamental link between the brain's intrinsic spatio‐temporal network structure and human cognitive function.

## 4 Materials and Methods

### 4.1 Participants and Dataset

We used data from the HCP 1200 Subjects Release (Van Essen et al. 2013) for our analyses. This comprehensive dataset includes protocols involving resting‐state fMRI, resting‐state MEG, and structural MRI (Barch et al. 2013; Larson‐Prior et al. 2013; Smith et al. 2013). For the primary fMRI and MEG connectivity analyses, we initially considered 95 participants who completed the MEG protocol. From this group, a subset of 55 individuals (26 female, aged 22–35) was selected to avoid family‐based confounds by including only one member from each twin pair, following procedures similar to Soyuhos and Baldauf (2023). For the subsequent CPM analyses, we used data from a larger cohort of 371 HCP participants (192 female, aged 22–36) selected from the HCP 1200 release such that each participant came from a different family structure and had both resting‐state fMRI and behavioral data for the face‐matching and scene *n*‐back tasks. All participants included in these analyses were healthy adults with no reported neurological or psychiatric disorders, and informed consent was obtained prior to participation. Further details on inclusion and exclusion criteria for the HCP cohort can be found in Van Essen et al. (2013). The anonymized dataset is available via ConnectomeDB (Hodge et al. 2016; Marcus et al. 2013).

### 4.2 Behavioral Measures

Behavioral data were derived from tasks administered as part of the HCP task fMRI battery (Barch et al. 2013). For this study, we focused on behavioral scores from a face‐matching task and a scene *n*‐back task. In the face‐matching task, participants viewed a target face presented above two alternative faces (Figure 4A). The faces displayed either an angry or fearful expression. Participants then indicated via button press which alternative (left or right) matched the target. The scene *n*‐back task alternated between 0‐back and 2‐back conditions, signaled by on‐screen prompts (Figure 4B). In the 0‐back condition, participants indicated via button press whether the currently displayed scene matched the predefined target shown for that task. In the 2‐back condition, participants indicated whether the current scene matched the one shown two images previously or not. During scanning, each participant completed 36 face‐matching trials and 20 scene trials in each of the 0‐back and 2‐back conditions (Barch et al. 2013). For the CPM analysis, we used the median RT from correct trials as the behavioral score, calculated separately for the face‐matching, 0‐back scene, and 2‐back scene tasks.

### 4.3 Acquisition and Preprocessing of Resting‐State fMRI and MEG Data

The fMRI data were obtained from the minimally preprocessed CIFTI dense time series available on ConnectomeDB to ensure data reproducibility (Glasser et al. 2013; Smith et al. 2013). Imaging was performed using a customized Siemens 3 T Connectome Skyra scanner equipped with a 32‐channel head coil and body transmission coil. T1‐weighted structural images were collected using a 3D MPRAGE sequence with 0.7 mm isotropic resolution (TR = 2400 ms; TE = 2.14 ms; TI = 1000 ms; flip angle = 8°). The data consisted of four runs, each approximately 15 min long. During acquisition, participants were instructed to maintain fixation on a crosshair presented on a dark background. Imaging parameters for fMRI included: TR = 720 ms, TE = 33.1 ms, flip angle = 52°, field of view (FOV) = 208 × 180 mm, matrix size = 104 × 90, slice thickness = 2 mm, and a multi‐band factor of 8. The HCP minimal preprocessing pipeline for fMRI involved correction for spatial distortions, head motion, and B0 field inhomogeneities. Functional images were registered to the T1‐weighted structural image and subsequently normalized to 2 mm MNI space. Following normalization, the functional data were resampled to a standard mesh and integrated into a standard gray ordinates space using MSMAll registration. This approach ensured consistent alignment of cortical and subcortical regions across participants, facilitating accurate group‐level analysis (Glasser et al. 2013; Smith et al. 2013).

The MEG data were collected using a Magnes 3600 whole‐head scanner featuring 248 magnetometers and 23 reference channels. Participants were recorded in a supine position while fixating on a red crosshair. Data were sampled at a rate of 2034.5101 Hz and stored in a 4D file format. Electrooculography, electrocardiography, and electromyography were recorded concurrently to aid in artifact correction. Preprocessing for MEG followed methods described by Soyuhos and Baldauf (2023). The Preprocessed Resting‐State package from ConnectomeDB provided annotations identifying bad channels, bad segments, and non‐brain artifact components (Hodge et al. 2016). Artifacts, such as those arising from eye blinks and muscle activity, were identified and corrected using functions within the FieldTrip toolbox (Oostenveld et al. 2011). The MEG data were then bandpass filtered between 1.3 and 150 Hz, and notch filters were applied to eliminate 60 Hz and 120 Hz power line noise.

### 4.4 Resting‐State fMRI and MEG Connectivity Analysis

To calculate functional connectivity from the fMRI data, we followed the steps outlined in Smith et al. (2013). First, the minimally preprocessed CIFTI dense time series were normalized ((*x* − mean)/stdev). Data from all four runs for each participant were then concatenated into a single continuous dataset (one hour per subject). Brain regions were defined using the HCP‐MMP1 atlas and parcel‐wise time series were extracted using the Connectome Workbench tools (Glasser et al. 2013; Smith et al. 2013). Functional connectivity matrices (360 × 360) were then computed from the parcel time series in MATLAB using the FSLNets toolbox, specifically the nets_netmats function with the ridgep option set to 0.01, which implements ridge‐regularized partial correlations rather than unregularized partial correlations. This regularized partial‐correlation approach was chosen because it aims to infer direct connections between node pairs by statistically removing the influence of time series from all other network nodes, while improving the stability of the estimated connectome in high‐dimensional settings (Peterson et al. 2025; Smith et al. 2013). Seed‐based exploratory and ROI‐based connectivity analyses were then performed separately for the left and right seed regions within their respective hemispheres.

For the MEG data, frequency‐specific functional connectivity matrices were calculated following the steps detailed in Soyuhos and Baldauf (2023). We began by reconstructing source‐level activity using the Brainstorm toolbox (Tadel et al. 2011), mapping the activity onto each subject's native cortical surface. Specifically, minimum‐norm estimation (MNE) was used to estimate cortical activity at 15,002 distributed sources across the cortex (Baillet et al. 2001; Dale et al. 2000; Hämäläinen and Ilmoniemi 1994). This source‐level data was then parcellated into the same 360 regions defined by the HCP‐MMP1 atlas (Glasser et al. 2016). To account for the distinct contributions of phase‐ and amplitude‐coupling to functional connectivity (Daffertshofer et al. 2018; Siems and Siegel 2020), we employed both phase‐ and amplitude‐based connectivity measures. Specifically, we used the imaginary part of coherency (Nolte et al. 2004) to assess phase relationships and the orthogonalized power envelope correlation (Hipp et al. 2012) to measure amplitude‐based connectivity. These metrics were selected for their effectiveness in reducing spatial leakage artifacts and enhancing the consistency of group‐level analyses (Bastos and Schoffelen 2016; Colclough et al. 2016; Duan et al. 2021).

Furthermore, to analyze dominant directional interactions between our seed regions (FFA, PPA) and target regions of interest (ROIs) during the resting state, we computed PDC (Baccalá and Sameshima 2001). PDC values were first calculated for each unidirectional connection between seed regions and target ROIs. Then, for each pair, the dominant direction of influence was determined by statistically comparing the PDC values for the opposing connections (Seed → ROI vs. ROI → Seed) to identify the significantly stronger direction. PDC was selected based on several advantages: it primarily considers direct interactions, is generally regarded as relatively insensitive to source leakage, and exhibits high group‐level repeatability compared to alternative directionality measures (Colclough et al. 2016). All MEG functional connectivity analyses were performed across distinct frequency bands, including delta (1–4 Hz), theta (4–8 Hz), alpha (8–13 Hz), beta (13–30 Hz), and gamma (30–100 Hz), to assess frequency‐specific interactions between regions.

### 4.5 Identifying Seed and Target ROIs

To determine the specific parcels in the HCP‐MMP1 atlas corresponding to our seed regions for the FFA and PPA, we first generated meta‐analytic association maps using Neurosynth (Yarkoni et al. 2011). These maps identified regions consistently activated across numerous studies: an analysis for the term “face” included 896 studies (31,842 activation foci), and an analysis for “place” included 189 studies (6595 activation foci). These Neurosynth association maps were generated in MNI152 2 mm space and represented statistically significant regions of consistent activation (FDR‐corrected, *q* < 0.01). We then used the AFNI toolbox (Cox 1996) to identify spatially contiguous clusters of these significant voxels using a nearest‐neighbor algorithm where voxels were grouped if their faces touched. The top two largest by volume (in voxels) for each map (“face” and “place”) were extracted. These clusters' MNI coordinates for both their peak activation (Peak; defined as the voxel with the highest statistical value within the cluster) and their center of mass (CMass; representing the average spatial location of the cluster) are presented in Table S1. MNI coordinates are reported in LPI order, where positive *X*, *Y*, and *Z* values correspond to anatomical Right, Anterior, and Superior, respectively. Subsequently, these extracted Peak and CMass MNI coordinates were localized by referencing them against the volumetric version of the HCP‐MMP1 atlas (Bedini et al. 2023).

The target ROIs were selected using a data‐driven approach designed to isolate areas exhibiting distinct functional connectivity profiles relative to the FFA and PPA seed regions. We performed statistical tests on subject‐specific fMRI partial correlation connectivity maps. Specifically, for each parcel in the MMP1 atlas within each hemisphere, we compared its connectivity strength to the ipsilateral FFA seed region versus its connectivity strength to the ipsilateral PPA seed region across subjects. Parcels exhibiting significantly stronger functional connectivity (*p* < 0.05, FDR‐corrected) with either the FFA seed or the PPA seed were selected as target ROIs for all subsequent analyses. This procedure identified 56 target ROIs in total across both hemispheres (detailed in Table 1), categorized based on their predominant functional link to either the face or scene processing networks.

In an additional control analysis, we also defined a second parahippocampal seed corresponding to a more anterior portion of PPA. Prior work has shown that PPA is organized along a posterior–anterior gradient, with a boundary around MNI *y* ≈−42 mm in 2 mm MNI space separating posterior from anterior PPA (Baldassano et al. 2016). Based on this, we identified the PHA2 parcel as the region immediately anterior to the PHA3 seed along the *Y*‐axis in the HCP‐MMP1 atlas. For this anterior seed, we repeated the same seed‐based connectivity and ROI selection procedures as for PHA3, contrasting the connectivity profiles of the FFA seed (FFC) with those of the PHA2 seed to identify parcels that were preferentially connected to each region.

### 4.6 Connectome‐Based Predictive Modeling

To assess whether individual differences in resting‐state functional connectivity could predict behavioral performance, we employed CPM analysis. CPM is a data‐driven, cross‐validated framework (Shen et al. 2017) that moves beyond simple correlation by building and rigorously testing models that predict individual behavioral scores from brain connectivity patterns. This approach provides a robust assessment of brain‐behavior relationships while mitigating the risk of overfitting common in non‐cross‐validated analyses (Finn et al. 2015; Rosenberg and Finn 2022; Shen et al. 2017).

We utilized the independent cohort of 371 participants described previously. Prior to modeling, specific exclusion criteria were applied. Eighteen subjects were excluded due to excessive head motion (mean framewise displacement > 0.15 mm). Subjects missing behavioral data for a specific task were further excluded from the corresponding model, resulting in final sample sizes of 350 subjects for the face‐matching task model and 352 subjects for the 0‐back and 2‐back scene task models. Additionally, we confirmed that residual head motion was not significantly correlated with the behavioral scores within each final sample (Spearman's rank correlation: Face‐matching task: *r* = 0.06, *p* = 0.26; 0‐back scene task: *r* = 0.03, *p* = 0.51; 2‐back scene task: *r* = 0.02, *p* = 0.77), ensuring that prediction performance was not driven by motion artifacts.

Separate CPM analyses were performed using network‐specific connectivity matrices as input. We defined two distinct networks based on the target ROI selection in Table 1. The first, a face processing network, comprised 37 regions total. This included the bilateral FFA seed regions plus the 35 ROI instances across both hemispheres identified in Table 1 as predominantly connected to the FFA seed. The second, a scene processing network, comprised 23 regions total. This included the bilateral PPA seed regions plus the 21 ROI instances across both hemispheres identified in Table 1 as predominantly connected to the PPA seed. We hereafter refer to these as the “FFA network” and “PPA network”, i.e., networks of parcels defined by their preferential fMRI connectivity with the FFA and PPA seeds, respectively. The input connectivity matrices for each subject were thus either 37 × 37 (FFA network) or 23 × 23 (PPA network), containing the fMRI partial correlations between the respective pairs of regions within that network. We used CPM to predict face‐matching RTs from the face processing network connectivity and to predict 0‐back and 2‐back scene RTs from the scene processing network connectivity. To test the specificity of these network‐behavior relationships, we also performed control analyses, predicting face‐matching RTs from the scene network and predicting both scene task RTs from the face network.

For each distinct CPM analysis, a leave‐one‐out (LOO) cross‐validation procedure was implemented across the respective final sample size. This involved iteratively training the model on N‐1 subjects and testing its predictive performance on the single held‐out subject, ensuring the independence between training and testing data at each fold. Within each training fold, several steps were performed following Shen et al. (2017). First, for feature selection, the strength of each edge (fMRI partial correlation value) within the relevant network matrix was correlated with the corresponding behavioral measure across the N‐1 training subjects using Spearman's rank correlation. Edges exhibiting a significant negative correlation with the behavioral score (RTs in this case; *p* < 0.05) were selected as predictive features. We focused specifically on negatively correlated edges as preliminary analyses indicated that positively correlated edges did not yield significant predictive models. Next, for feature summarization, a single summary score was calculated for each subject in the training set by summing the fMRI partial correlation values of all edges selected in the previous step. Finally, for model building, a simple linear regression model (*y* = *mx* + *b*) was fitted to the training data, predicting the behavioral scores (*y*) from the calculated summary scores (*x*). The model built in each training fold (i.e., the fitted slope “*m*” and intercept “*b*”) was then applied to the left‐out test subject. First, the summary score for the test subject was calculated using the same feature mask (set of predictive edges) defined from the training fold. Then, this summary score was input into the linear model derived from the training fold to generate the predicted behavioral score for that test subject. After completing all LOO folds for a given analysis, overall model performance was evaluated by calculating the Spearman's rank correlation (*r*) between the predicted scores and the true observed scores across all subjects.

We then performed two additional sets of control analyses to clarify the network‐level basis of the observed predictions. First, we repeated the procedure using “seed‐excluded” versions of the face and scene networks (Figure S5). For each subject, we derived modified connectivity matrices from the original 37 × 37 and 23 × 23 networks in which all connections involving the FFA or PPA seed parcels, respectively, were removed before feature selection. CPM was then applied to these seed‐excluded networks using the same LOO cross‐validation and permutation‐testing framework to assess whether predictive information was distributed across non‐seed regions within each network.

Second, to assess the behavioral relevance of posterior versus anterior PPA networks, we repeated the CPM analysis using the scene network defined from the PHA2‐based connectivity contrast. As in the main analysis, we constructed a network from all parcels showing preferential connectivity with the PHA2 seed and used the corresponding fMRI partial correlations to predict median RTs in the 0‐back and 2‐back scene tasks. The same LOO cross‐validation and permutation‐testing procedures described above were applied to this PHA2‐anchored scene network.

### 4.7 Statistical Analysis

Statistical analyses were performed using the Statistics and Machine Learning Toolbox in MATLAB R2023a and the boot package in R (version 4.4.1). For the fMRI and MEG connectivity analyses comparing connectivity strengths, we used non‐parametric two‐sided Wilcoxon signed‐rank tests. The significance threshold (alpha level) was set at *p* < 0.05. Corrections for multiple comparisons across parcels were applied using the false discovery rate (FDR) procedure (Benjamini and Hochberg 1995), with a corrected significance threshold of *q* < 0.05. For statistically significant target regions, *z*‐scores were calculated from the adjusted *p*‐values to quantify differences in connectivity strength. For the CPM analysis, the statistical significance of the prediction accuracy (Spearman correlation between predicted and observed scores) for each model was assessed non‐parametrically using permutation testing (1000 iterations). In each permutation, the behavioral scores were randomly shuffled across subjects, the entire LOO CPM procedure was repeated using the shuffled scores, and the resulting correlation between predicted and shuffled scores was stored. This process built an empirical null distribution of correlation coefficients expected by chance. The final *p*‐value for the actual model performance was calculated using a right‐tailed (one‐sided) test as the proportion of permutations that yielded a correlation coefficient greater than or equal to the correlation obtained with the true, unshuffled behavioral scores. Confidence intervals for Spearman correlation coefficients were estimated in R using non‐parametric bootstrap resampling (5000 iterations) with bias‐corrected and accelerated (BCa) intervals, implemented in the boot package.

## Author Contributions

**Daniel Baldauf**, **Aurelia Scarpa**, and **Orhan Soyuhos:** conceptualization. **Orhan Soyuhos**, **Aurelia Scarpa**, and **Daniel Baldauf:** methodology. **Orhan Soyuhos:** software. **Orhan Soyuhos** and **Aurelia Scarpa:** formal analysis. **Orhan Soyuhos:** visualization. **Orhan Soyuhos** and **Aurelia Scarpa:** writing – original draft. **Orhan Soyuhos** and **Daniel Baldauf:** writing – review and editing. **Daniel Baldauf** and **Orhan Soyuhos:** funding acquisition.

## Funding

This work was supported by the National Science Foundation under the NSF NRT NeuralStorm program (Award No. 2152260).

## Disclosure

Our article reports human subjects. Recruitment meets scientific requirements & HBMs expectation of inclusivity.

## Conflicts of Interest

The authors declare no conflicts of interest.

## Supporting information


**Data S1:** Supporting Information.

## Data Availability

The data for the current study were sourced from the 1200 Subjects Release of the Human Connectome Project (Larson‐Prior et al. 2013; Van Essen et al. 2013). These data are publicly accessible via ConnectomeDB (db.humanconnectome.org; Hodge et al. 2016).
