# Supplementary material for: Distinct Resting‐State Connectomes for Face and Scene Perception Predict Individual Task Performance
Source: Hum Brain Mapp. 2026 Mar 23;47(5):e70498. doi: 10.1002/hbm.70498 (PMC13081693; doi:10.1002/hbm.70498)
Supplement: Supplementary file 1 — Data S1: Supporting Information. [file HBM-47-e70498-s001.pdf]

Distinct resting-state connectomes for face and scene perception predict individual task performance

Orhan Soyuhos[1,2,3], Aurelia Scarpa[1,4], and Daniel Baldauf[1,*]

[1]Centre for Mind/Brain Sciences (CIMeC), University of Trento, Trento, Italy
[2]Center for Neuroscience, University of California, Davis, Davis, CA, USA
[3]Department of Psychology, University of California, Davis, Davis, CA, USA
[4]Research Group Health Psychology, Faculty of Psychology and Educational Sciences, KU Leuven, Belgium
[*]Correspondence: daniel.baldauf@unitn.it

Supplementary information

Figures

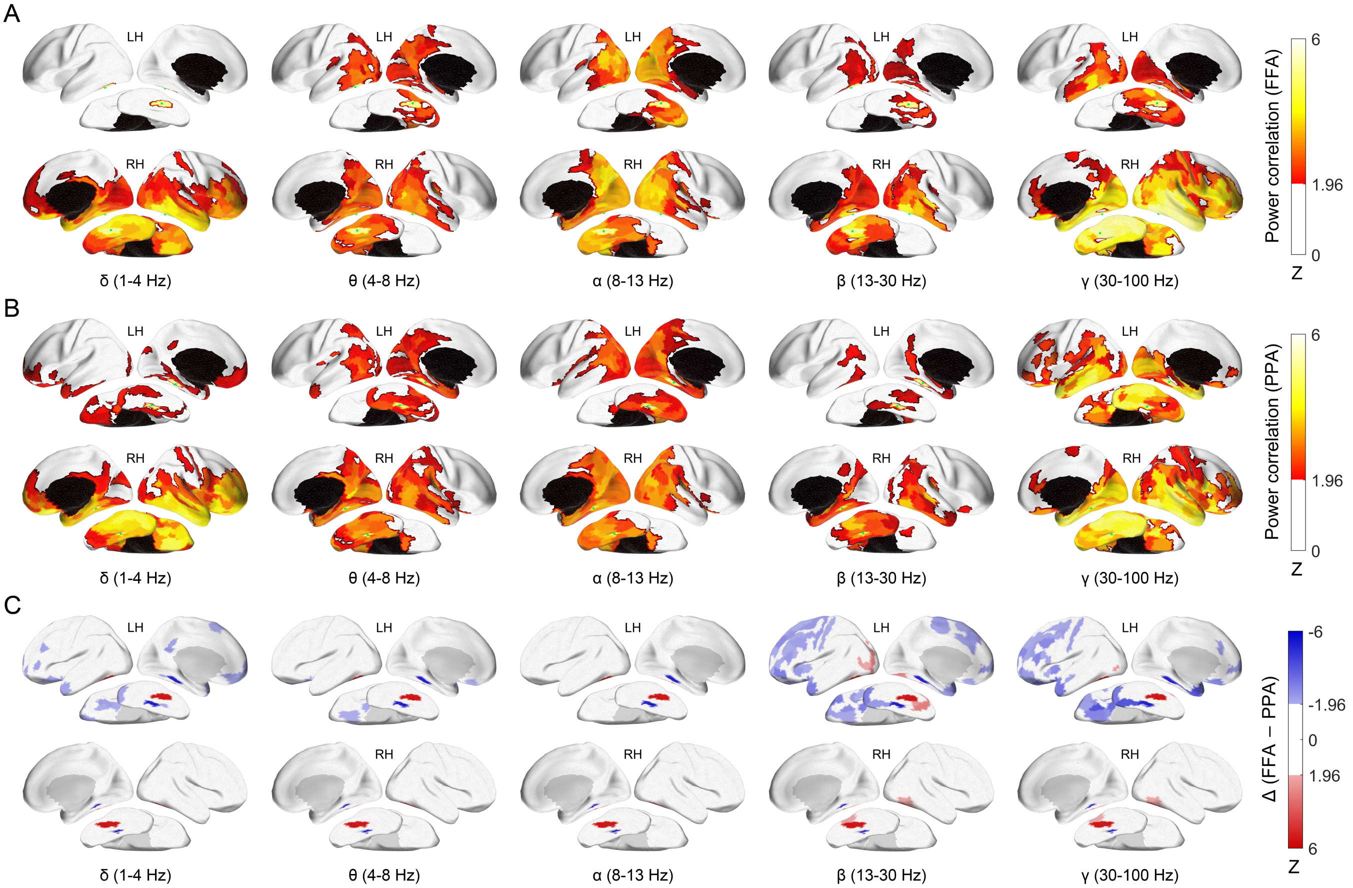

Figure S1 | MEG power envelope correlation maps for the Fusiform Face Area (FFA) and Parahippocampal Place Area (PPA).

(A) Whole-brain frequency-specific functional connectivity maps for the FFA based on the orthogonalized power envelope correlation method. The green dot indicates the FFA seed region. Functional connectivity is displayed for the left (LH) and right (RH) hemispheres on each ipsilateral side. (B) Same as (A) but for the PPA. (C) Direct comparison of whole-brain functional connectivity between the FFA (red) and PPA (blue), highlighting areas with preferential connectivity to each region. Statistical significance was assessed using a $p < 0.05$ threshold, FDR-corrected for 180 ROIs, and represented as z-scores.

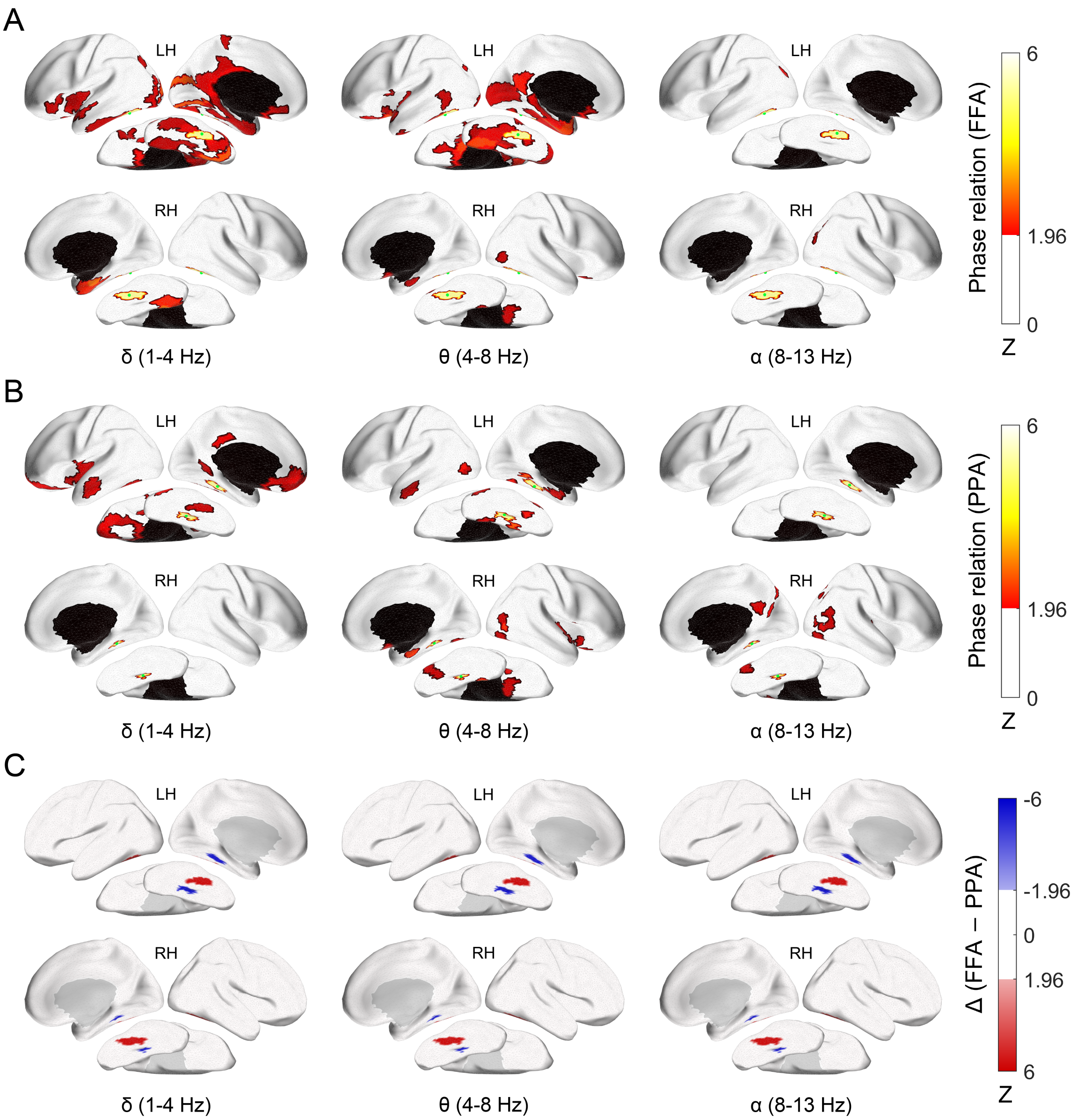

Figure S2 | MEG phase connectivity maps for the Fusiform Face Area (FFA) and Parahippocampal Place Area (PPA).

(A) Whole-brain frequency-specific functional connectivity maps for the FFA based on the imaginary part of the coherency method. The green dot indicates the FFA seed region. Functional connectivity is displayed for the left (LH) and right (RH) hemispheres on each ipsilateral side. (B) Same as (A) but for the PPA. There are no significant regions in beta and gamma frequency bands. (C) Direct comparison of whole-brain functional connectivity between the FFA (red) and PPA (blue), highlighting areas with preferential connectivity to each region. There were no significant areas that have predominant connectivity with either FFA or PPA across all frequency bands. Statistical significance was assessed using a $p < 0.05$ threshold, FDR-corrected for 180 ROIs, and represented as z-scores.

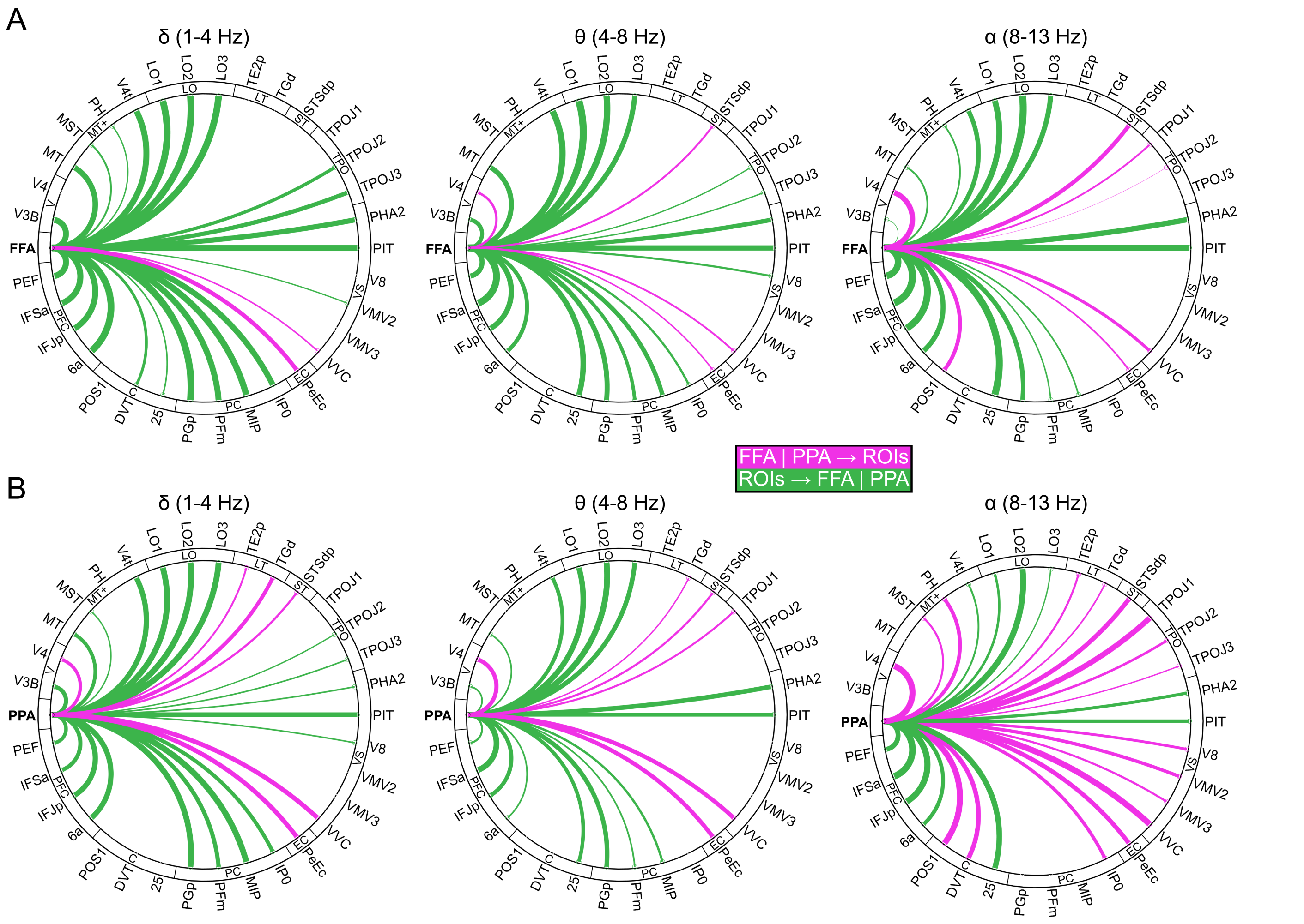

Figure S3 | Frequency-specific directional connectivity analyses of the Fusiform Face Area (FFA) and Parahippocampal Place Area (PPA).

(A) Circular graphs show the directional connectivity between the FFA and the brain regions listed in Table 1 across both hemispheres. Magenta lines indicate the directions of interaction from the FFA to the regions of interest (ROIs), while green lines indicate the directions of interaction from the ROIs to the FFA. (B) Same as (A) but for the PPA. Statistical significance was assessed using a $p < 0.05$ threshold, FDR-corrected for 33 ROIs, and represented as z-scores. The width of the lines reflects the strength of the connectivity. Abbreviations for the ROI groupings shown in the figure include: V = Visual areas, MT+ = Middle temporal complex, LO = Lateral occipital areas, LT = Lateral temporal cortex, ST = Superior temporal sulcus, TPO = Temporoparietal junction, VS = Ventral visual stream, EC = Entorhinal cortex, PC = Parietal cortex, C = Cingulate cortex, and PFC = Prefrontal Cortex.

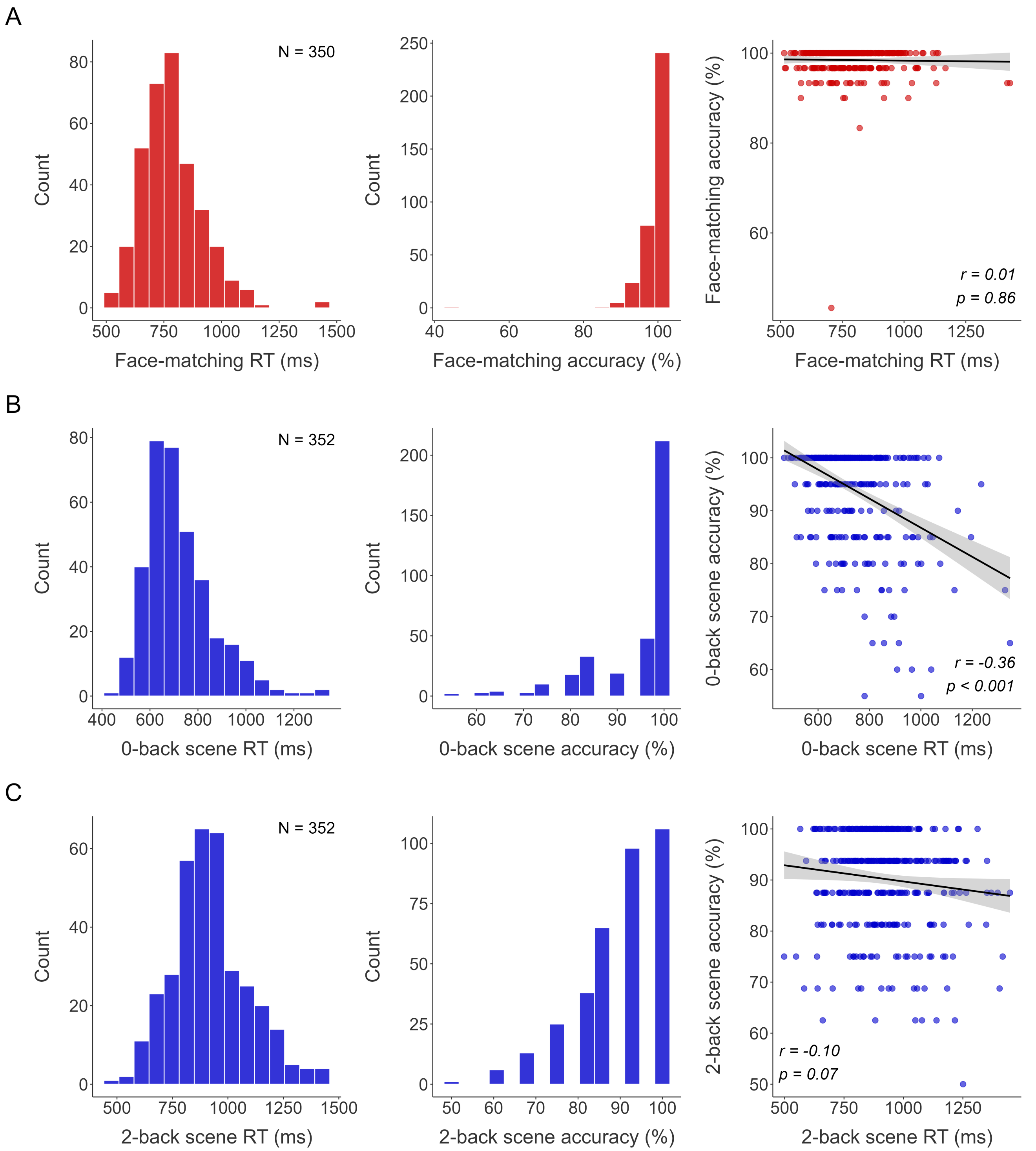

Figure S4 | Reaction times and accuracies for the face-matching and n-back scene tasks.

(A) Distributions of median reaction times (RTs; left), accuracies (middle), and their relationship (right) across participants for the face-matching task (N = 350). Median RTs were computed from correct trials. The scatter plot shows accuracy as a function of median RT, with a linear fit (black line) and 95% confidence interval (gray band). Spearman's rank correlation coefficient (r) and p-value are indicated in the panel. (B) and (C) Same as (A), but for the 0-back and 2-back scene tasks, respectively (N = 352).

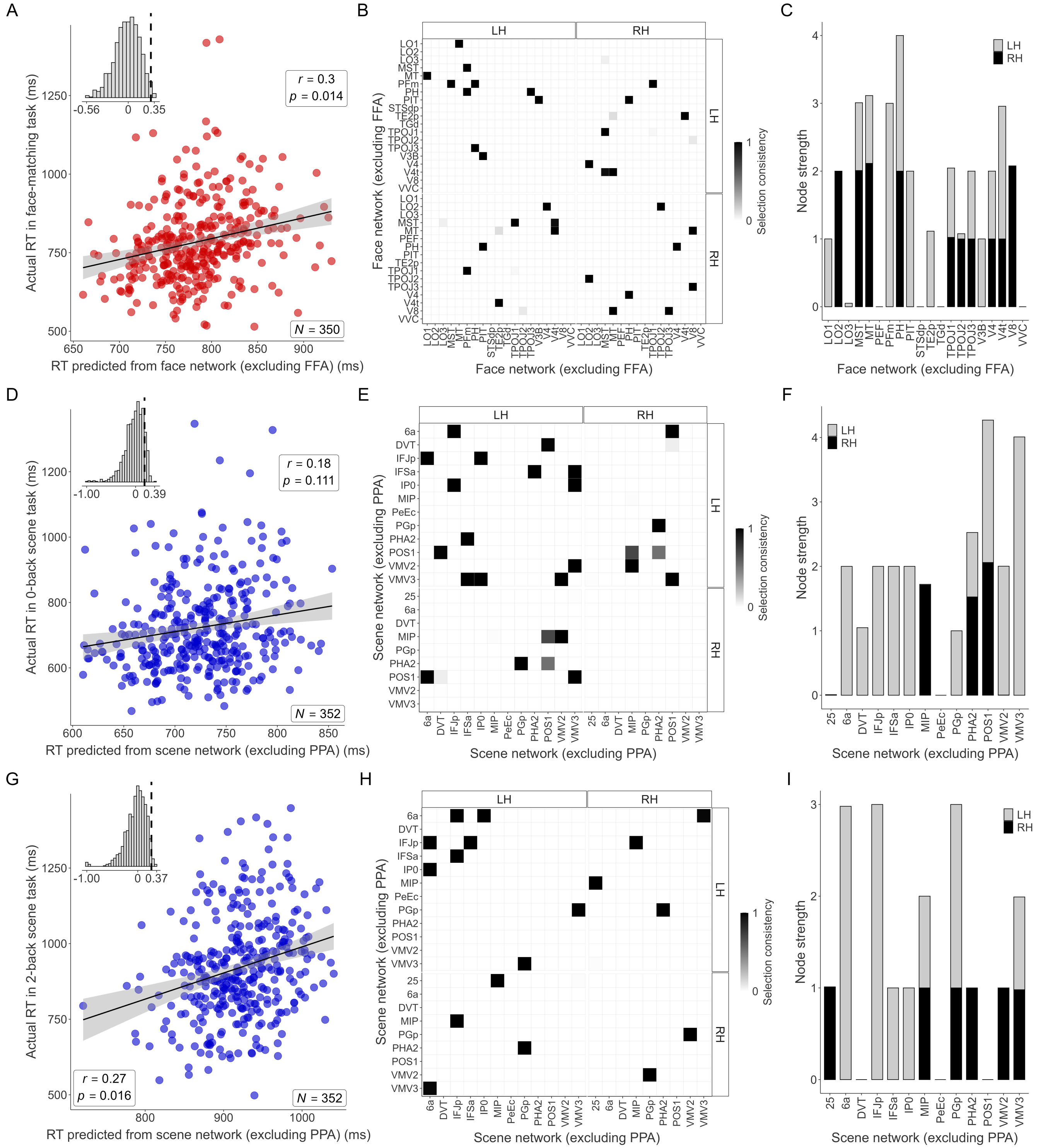

Figure S5 | Connectome-based predictive modeling (CPM) of median reaction times using face and scene networks excluding the FFA/PPA seeds.

(A) Scatter plot comparing actual and predicted median reaction times (RTs; ms) for the face-matching task, based on CPM using resting-state fMRI connectivity within the face network (excluding FFA). This network is derived from the original FFA-anchored network used in the main analyses, after removing all connections that include the FFA seed. The black line shows the linear regression fit and the gray shading indicates the 95% confidence interval. The inset histogram shows the permutation-based null distribution of correlations, with the observed correlation marked by a dashed vertical line. (B) Scatter plot comparing actual and predicted median RTs for the 0-back scene task, based on CPM using resting-state

fMRI connectivity within the scene network (excluding PPA). This network is derived from the original PPA-anchored network, after removing all connections that include the PPA seed. The regression line, confidence interval, and permutation-based null distribution are shown as in (A). (C) Scatter plot comparing actual and predicted median RTs for the 2-back scene task using the same scene network (excluding PPA). The regression fit, 95% confidence interval, and permutation-based null distribution are displayed as in (A) and (B).

Tables

Volume	Peak			Center of Mass	
(voxels)	(z-score)	XYZ (MNI)	HCP-MMP1	XYZ (MNI)	HCP-MMP1
**‘Face’ association test**					
4706	21.0	+40.0 -50.0 -20.0	Right FFC	+42.3 -59.7 -13.3	Right PH
3217	17.4	-40.0 -52.0 -22.0	Left FFC	-37.8 -65.5 -13.7	Left FFC
**‘Place’ association test**					
481	11.0	-28.0 -46.0 -10.0	Left PHA3	-29.7 -45.6 -10.3	Left PHA3
361	8.0	+28.0 -44.0 -14.0	Right VVC	+29.7 -43.0 -11.7	Right PHA3

Table S1 | Principal clusters from ‘Face’ and ‘Place’ Neurosynth association test maps.

This table summarizes the two largest activation clusters derived from Neurosynth term-based meta-analytic association maps for the terms ‘Face’ and ‘Place’ (input maps were thresholded at a False Discovery Rate of $q < 0.01$). For each reported cluster, the table lists its volume (in voxels), the peak z-score, the MNI coordinates (X,Y,Z) of both the peak activation and the center of mass, and their respective anatomical localizations within the Human Connectome Project Multi-modal Parcellation (HCP-MMP1) atlas. MNI coordinates are reported in LPI order.

Parcellation	Brain Region	Left Hemisphere		Right Hemisphere	
		P-value	Z-score	P-value	Z-score
A4	Auditory Cortex	<0.01	2.37	–	–
FST	Fundus of Superior Temporal (MT+ Complex)	–	–	<0.05	2.05
LIPd	Lateral Intraparietal Area	<0.05	2.07	–	–
LO1	Lateral Occipital Area	<0.01	2.84	<0.001	3.79
LO2	Lateral Occipital Area	<0.001	5.12	<0.001	4.82
LO3	Lateral Occipital Area	<0.001	4.39	<0.001	4.68
MST	Medial Superior Temporal (MT+ Complex)	<0.001	4.03	<0.001	3.80
MT	Middle Temporal (MT+ Complex)	<0.001	5.70	<0.001	4.12
PEF	Premotor Eye Field	–	–	<0.01	2.35
PH	Area PH (MT+ Complex)	<0.001	5.90	<0.001	5.91
PIT	Posterior Inferior Temporal Complex	<0.001	5.90	<0.001	5.91
PeEc	Perientorhinal and Ectorhinal Complex	<0.01	2.78	<0.01	2.73
ProS	ProStriate Area	–	–	<0.01	2.35
STSva	Superior Temporal Sulcus	<0.01	2.42	–	–
TE1m	Middle Temporal Cortex	–	–	<0.01	2.43
TE2p	Inferior Temporal (IT) Cortex	<0.001	5.90	<0.001	5.91
TF	Inferior Temporal (IT) Cortex	<0.001	5.59	<0.001	5.79
TGd	Temporal Polar Cortex	<0.001	3.26	–	–
TPOJ1	Temporo-Parieto-Occipital Junction	<0.001	3.54	<0.001	5.02
TPOJ2	Temporo-Parieto-Occipital Junction	<0.001	4.96	<0.001	5.10
TPOJ3	Temporo-Parieto-Occipital Junction	<0.001	4.85	<0.001	5.07
V4	Visual Area V4	<0.001	3.54	<0.01	2.44
V4t	Area V4t (MT+ Complex)	<0.001	5.55	<0.001	5.79
V8	Visual Area V8 (Part of Ventral Stream)	<0.001	5.90	<0.001	5.91
VMV2	Ventral Medial Visual Area	–	–	<0.05	2.08
VVC	Ventral Visual Complex	<0.001	5.90	<0.001	5.91

Table S2 | Brain regions exhibiting significant functional connectivity with the Fusiform Face Area (FFA).

The table lists areas from the Human Connectome Project multi-modal parcellation (HCP-MMP1) atlas that have significant functional coupling with the FFA (‘Parcellation’ column; Figure 1B). It also provides an approximate functional label for each region based on Glasser et al. (2013) (‘Brain Region’ column). Columns display the statistical significance (P-value) and strength (Z-score) of the functional connectivity, presented separately for the left (LH) and right (RH) hemispheres. Dashes (–) indicate that significant connectivity was not detected for that region in the specified hemisphere.

Parcellation	Brain Region	Left Hemisphere		Right Hemisphere	
		P-value	Z-score	P-value	Z-score
6a	Superior Premotor Cortex	–	–	<0.05	2.18
DVT	Posterior Cingulate Cortex	<0.001	4.93	<0.001	4.95
H	Part of Hippocampus	<0.05	1.74	–	–
IP0	Inferior Parietal Cortex	<0.001	4.27	<0.001	3.63
PGp	Inferior Parietal Cortex	<0.001	5.76	<0.001	5.64
PH	Area PH (MT+ Complex)	<0.001	5.45	–	–
PHA2	Parahippocampal Area	<0.001	5.83	<0.001	5.87
POS1	Posterior Cingulate Cortex	<0.01	2.63	<0.001	4.46
PeEc	Perientorhinal and Ectorhinal Complex	<0.001	5.80	<0.001	3.15
TE2p	Lateral Temporal Cortex	<0.05	2.05	–	–
TF	Lateral Temporal Cortex	<0.001	5.24	<0.001	5.87
VMV2	Ventral Medial Visual Area	<0.001	5.83	<0.001	5.87
VMV3	Ventral Medial Visual Area	<0.001	4.58	<0.001	4.33
VVC	Ventral Visual Complex	<0.001	5.83	<0.001	5.87

Table S3 | Brain regions exhibiting significant functional connectivity with the Parahippocampal Place Area (PPA).

The table lists areas from the Human Connectome Project multi-modal parcellation (HCP-MMP1) atlas that have significant functional coupling with the PPA (‘Parcellation’ column; Figure 1C). It also provides an approximate functional label for each region based on Glasser et al. (2013) (‘Brain Region’ column). Columns display the statistical significance (P-value) and strength (Z-score) of the functional connectivity, presented separately for the left (LH) and right (RH) hemispheres. Dashes (–) indicate that significant connectivity was not detected for that region in the specified hemisphere.
